# Geomagnetism-Aided Indoor Wi-Fi Radio-Map Construction via Smartphone Crowdsourcing

**DOI:** 10.3390/s18051462

**Published:** 2018-05-08

**Authors:** Wen Li, Dongyan Wei, Qifeng Lai, Xianghong Li, Hong Yuan

**Affiliations:** 1 Academy of Opto-Electronics, Chinese Academy of Sciences, Beijing 100094, China; wen.li@aoe.ac.cn (W.L.); laiqifeng@aoe.ac.cn (Q.L.); lixianghong@aoe.ac.cn (X.L.); yuanh@aoe.ac.cn (H.Y.); 2 University of Chinese Academy of Sciences, Beijing 100049, China

**Keywords:** indoor localization, Wi-Fi fingerprint, crowdsourcing data, magnetic field, radio-map construction

## Abstract

Wi-Fi radio-map construction is an important phase in indoor fingerprint localization systems. Traditional methods for Wi-Fi radio-map construction have the problems of being time-consuming and labor-intensive. In this paper, an indoor Wi-Fi radio-map construction method is proposed which utilizes crowdsourcing data contributed by smartphone users. We draw indoor pathway map and construct Wi-Fi radio-map without requiring manual site survey, exact floor layout and extra infrastructure support. The key novelty is that it recognizes road segments from crowdsourcing traces by a cluster based on magnetism sequence similarity and constructs an indoor pathway map with Wi-Fi signal strengths annotated on. Through experiments in real world indoor areas, the method is proved to have good performance on magnetism similarity calculation, road segment clustering and pathway map construction. The Wi-Fi radio maps constructed by crowdsourcing data are validated to provide competitive indoor localization accuracy.

## 1. Introduction

Location-based service (LBS) is some of the most important content that provides convenient and precise services for users, such as navigation for pedestrians or cars, mobile payment, taxi finding, bicycle sharing, intelligent guiding, loss prevention and so on. The global navigation satellite system (GNSS) can provide positioning services in most outdoor environments, however in the indoor environment, such as a big shopping mall, underground parking and museums, other positioning techniques should be considered due to the signal occlusion problem of GNSS. Currently, the indoor positioning techniques commonly used in consumer applications mainly include Wi-Fi signal strength fingerprint methods [1,2], range measurement methods using wireless signal [3,4], geomagnetic field matching methods [5,6], dead reckoning (DR) methods based on smartphone-mounted micro electro mechanical system (MEMS) [7,8] and localization using context recognition and landmarks [9,10]. All the mentioned localization methods have their own advantages and disadvantages, but the Wi-Fi fingerprint method usually plays the main role in indoor localization, for the reason that Wi-Fi has been deployed in almost all public places and its received signal strength (RSS) has good ability in location differentiation within a floor or between distinct floors.

Fingerprint-based Wi-Fi indoor localization system always consist of two phases: offline radio map construction and online localization by fingerprint matching. In the offline phase, RSS from Wi-Fi access points (APs) are labeled using the location coordinates of each reference point (RP), and all the RP data are stored together as a fingerprint database. In the online phase, user’s real time RSS measurements are matched to the fingerprint database using an algorithm, and the position with most similar RSS measurements is given as final position solution. To realize the offline phase, it is ideal that the RSS of all the detected APs be carefully calibrated to a location grid which completely covers the indoor map, and then the Wi-Fi radio map can be constructed exactly. However, this method is time-consuming and labor-intensive, as it needs repeated measurements on each RP to obtain statistical value of RSS and professional work to realize exact calibration of the indoor coordinates. Another challenge is that the constructed radio map would lose stability and even become invalid in some situations like fluctuating air humidity and indoor architecture changes [11]. In order to solve the above problems, some new kind Wi-Fi fingerprint construction techniques have been proposed, including data collection with the help of volunteers [12], simultaneous localization and mapping (SLAM) using Wi-Fi signal strength [13], RSS prediction based on exist fingerprints [14], and fingerprint construction using passive crowdsourcing data [15].

Redpin [16] and OIL [12] are previously proposed fingerprint-based indoor localization systems that omit the time-consuming training phase, in which the volunteer users report their current locations with room-level accuracy and corresponding Wi-Fi signal-strength. The more users that are uploading fingerprints at different locations, the more areas the database can cover. Aiming for a more flexible and scalable point in the design space of user-generated Wi-Fi localization system, Molé [17] arranges places in a hierarchy instead of an accurate map for floor plan and labels users fingerprints by semantic locations. These solutions depend on the active participation of users that results in inconveniences for the user experience and unknown measurement noises [18].

Ferris et al. [13] and Huang et al. [19] proposed signal-strength-based SLAM techniques. Reference [13] uses a Gaussian Process Latent Variable Model (GPLVM) to determine the latent-space locations of unlabeled signal strength data. Reference [19] presents a GraphSLAM-like algorithm for signal strength SLAM, which is viable for a broader range of environments due to its lack of special constraints and reduction of runtime complexity. WiFi-SLAM serves a promising solution for the problem of collection and maintenance of WiFi sensor models for large-scale localization, but it needs user trajectories with some closed loops and requires more calculation.

HIWL [20] uses K-means to produce discrete signal observation sequences, and train Hidden Markov Model (HMM) parameters by limited topology information of indoor environments. Through HMM training, the system learns the mapping relationship in geographical and signal distribution, and then matches the unlabeled fingerprints to the corresponding physical locations based on the mapping model. UMLI [21] proposes using nonparametric clustering methods to classify the unlabeled signal observations through two classification layers. By utilizing the clustering method, unlabeled signal data are classified into locations or rooms having similar RSS, achieving less site survey and labeling work. Chang et al. [14] developed a Minimum Inverse Distance (MID) algorithm to build a virtual database with uniformly distributed virtual RPs. The Local Gaussian Process (LGP) is then applied to estimate the virtual RPs’ RSSI values based on the crowdsourcing surveyed data. These methods need a set of surveyed fingerprints for model training and RSS prediction, so the quantity and quality of labeled data will seriously impact their accuracy.

With the popularization of smartphones and the increasing number of location-based service (LBS) users, research on passive methods of Wi-Fi radio map construction based on crowdsourcing has become a research hot spot currently. Its greatest advantage is that the crowdsourcing data can be obtained passively through user’s common behaviors when they use smartphone applications (APP). Meanwhile, the Wi-Fi radio map can be formed automatically and is easy to update thanks to the continuous uploading of user data.

WILL [22] is proposed as a wireless indoor logical localization approach that achieves room level location accuracy without site surveys. By exploiting user motions from mobile phones, independent radio signatures are previously connected following certain semantic rules to make a logical floor plan, and finally mapping it with a physical floor plan. Wang et al. [23] proposed an indoor subarea localization scheme which first constructs subarea fingerprints from crowdsourced RSS measurements using RL-clustering and then matches them to indoor layouts. They also proposed an online localization algorithm to deal with the device diversity issue. Both the WILL and Wang proposed methods divide user data into some clusters by RSS similarity and match them to subareas in a floor plan map. They can only provide room level localization rather than continuous positioning when a user walks in the indoor environment. Other systems use DR to compute user trajectories and furthermore label fingerprints in the indoor map pathway, which can provide a continuous localization in the indoor corridors (roads). Zee [24] use the inertial sensors present in the mobile phone to track them by a motion estimator (e.g., step counter, heading offset and stride length estimator) in an indoor environment, and implements an augmented particle filter both using floor map showing the pathways (e.g., hallways) and barriers (e.g., walls) and Wi-Fi scans to acquire crowdsourcing users’ position. RACC [25] identifies the indoor anchors (doors) as reference positions of the whole radio-map, in which doors’ RSS fingerprints can be identified according to motion detection when users walk through doors annotated with their corresponding physical locations by an adjacent recursive matching method. RCILS [26] detects people’s activities and trajectories in the indoor environment and matches them to a semantic graph of the indoor map using HMM. Then RSS collected along the trajectories can be labeled with location information. The air pressure detected by the barometer is used for elevator/stairs detection. RCILS also propose a trajectory fingerprint-based method in the online localization phase, which performs better when longer trajectory window is used in the sequence matching. Zee, RACC and RCILS can provide an accurate location solution in about 1~2 m, but they need an exact floor layout. PiLoc [15] divides a user’s single trajectory into disjoint path segments (turns and long straight lines) by detecting steps and turns, and utilized movement displacement (distance and direction) as well as the associated Wi-Fi signal to match path segments. Then PiLoc merges the clustered path segments annotated with displacement and signal strength information to derive a floor plan of walking paths annotated with radio signal strengths. It does not require manual calibration, prior knowledge and infrastructure support. A comparison of fingerprint localization methods without site survey via passive crowdsourcing data is shown in Table 1.

In addition, localization based on geomagnetic field has attracted attention in the indoor localization area for the fact it does not need infrastructure support and its stability compared to Wi-Fi signals. GROPING [27] utilizes magnetic fingerprinting collected by crowdsensing users and construct a floor map from an arbitrary set of walking trajectories. Map explorer records magnetic fields using smartphones and tags road junctions manually to help GROPING partition trajectories into segments. The similarity of magnetic intensity sequences is used to infer the overlapping segments among trajectories and stick them together. Reference [28] presents a SLAM algorithm based on measurements of the ambient magnetic field strength (MagSLAM) for pedestrians with foot-mounted sensors. Reference [29] considers magnetic field SLAM exploration using a mobile robot with a magnetometer and wheel encoders.

Different from the above methods, we propose in this paper a geomagnetism-aided indoor radio-map construction method via passive smartphone crowdsourcing. The proposed method don’t need exact floor layouts, but rather utilizes crowdsourcing traces to form the pathway map of a floor plan with merged Wi-Fi radio signal strengths annotated on it. It recognizes road segments from crowdsourcing traces by a cluster based on magnetism sequence similarity, which can be calculated exactly by a proposed feature matching algorithm even though the walking speeds may be different for each user.

The rest of the paper is organized as follows: Section 2 describes the opportunity and challenge in this problem and gives an algorithm overview. The algorithm details and experimental results are respectively given in Section 3 and Section 4. Finally, conclusions are presented in Section 5.

## 2. Problem and Algorithm Overview

### 2.1. Opportunity and Challenge

Currently, sensors mounted in smartphones are abundant, and their performance is better. The sensors mainly utilized in indoor localization are accelerometers, gyroscopes, magnetometers, electronic compasses, barometers and Wi-Fi, which provide acceleration, angular velocity, magnetic field, orientation, barometric pressure and Wi-Fi RSS. Most of the sensors measurements can be uploaded by users via smartphone APPs that provide payment, navigation, map or shop information services in indoor environments, called crowdsourcing data. Based on these data, the Wi-Fi radio map of the indoor environment can be constructed. The main components for indoor radio map construction via crowdsourcing data are users’ trajectory tracking and Wi-Fi fingerprint labeling to accurate locations. In the following content, we discuss the opportunities and challenges on the resources and techniques available in our problem. It mainly covers pedestrian dead reckoning (PDR) and fingerprint localization.

#### 2.1.1. Pedestrian Dead Reckoning

PDR is a technique which estimates relative locations and follow the tracks of a pedestrian via step detection, stride length estimation and heading determination. It is widely used in indoor localization systems with or without crowdsourcing, for its autonomy in pedestrian localization. In a smartphone-based PDR system, the accelerometer, gyroscope and magnetometer embedded with the smartphone are generally utilized to realize the PDR algorithm. 3-axis acceleration can be used to detect walking steps and estimate stride length. Besides, 3-axis angular velocity and magnetic field data are usually fused to determine the heading of pedestrians. There are some key challenges in this technique, which are addressed as follows [8,30]:

- The unconstrained human motions and smartphone postures make it complex to capture user’s motion modes, detect steps and estimate accurate walking orientation.
- Long/short term drift of accelerometer and gyroscope, which perform worse due to the low-cost IMU sensors embedded in smartphones, as well as magnetic disturbances result in inaccurate stride length estimation and heading determination.
- As a result of the above-mentioned two issues, the PDR localization output calculated by Equation (1) would not converge since its lacks an efficacious reference source:
  (1) $$\{\begin{array}{c} x_{t+1}=x_{t}+L_{t+1}\cdot \sin\phi_{t+1}\\ y_{t+1}=y_{t}+L_{t+1}\cdot \cos\phi_{t+1} \end{array}$$

Consequently, it is hard to use PDR directly for users’ trajectory tracking by crowdsourcing data. However, it still can benefit for distance estimation approximately in a limited area.

#### 2.1.2. Fingerprint

The Wi-Fi fingerprint on a location point is a vector of the RSS from all the scanned Wi-Fi APs at a same time. For different locations in indoor areas, the detected Wi-Fi RSS vectors are correspondingly different, due to the diversity of signal attenuation caused by different ranges, wall blocks and multipath from each APs to the smartphone. Meanwhile, the Wi-Fi RSS vectors are similar for adjacent locations. The location correlation of Wi-Fi fingerprint makes it available in indoor positioning. Similarly, the Bluetooth fingerprint and magnetic field fingerprint can also be used in the indoor localization based on the same principle as Wi-Fi fingerprints, and they are all available in crowdsourcing, so we will talk about them together and compare their advantages and disadvantages. For the reasons that the Bluetooth fingerprint has almost same features as the Wi-Fi fingerprint as they are all based on the wireless electromagnetic signal of similar frequency (2.4 GHz), and Bluetooth launchers are always few in most public indoor environments for their lack of internet access functions compared with Wi-Fi, so in this section we will focus on discussion of Wi-Fi fingerprints and magnetic fields, and furthermore show the opportunities and challenges of using them for indoor localization.

(1) Fingerprint Stability

**Figure 1:**
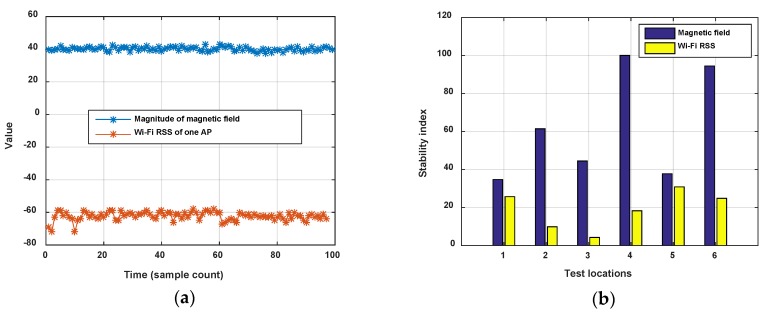
Comparison of stability between Wi-Fi and magnetic field at a simple location: (**a**) Time sequences for magnitude of magnetic field and Wi-Fi RSS of one AP measured on location 1 at the same time; (**b**) Stability index comparison between Wi-Fi and magnetic field for six test locations.

Stability comparisons between Wi-Fi and magnetic fingerprints have been given in some works. Here we adopt the *stability index* proposed in [27] to judge the stability between Wi-Fi RSS and magnetic field intensity. *Stability index* is the mean-to-standard deviation ratio of intensity, like Signal-to-Noise Radio (SNR). The Wi-Fi RSS of a settled AP and the magnitude of magnetic field are detected at a same location point and compared using the *stability index*. We detect six location points in an office room and corridor (staying for 5 min on each point), and calculate the *stability index* of the Wi-Fi and magnetic field signals. Figure 1 shows the result. It is seen that the magnetic field is obviously more stable than the Wi-Fi RSS.

**Figure 2:**
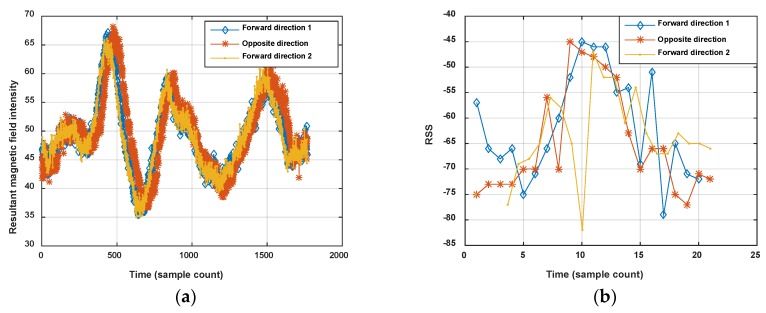
Comparison of the stability between Wi-Fi and magnetic field in a corridor: (**a**) Time sequences for the magnitude of the magnetic field generated in a same corridor using forward and opposite directions; (**b**) Time sequences for Wi-Fi RSS of one AP generated in the same corridor using forward and opposite directions. The sequences generated in opposite directions have been reversed in these two figures.

In addition, it is known that the 2.4 GHz Wi-Fi signal is easily absorbed by the human body, which leads to obvious changes of RSS with or without ambient crowds. Figure 2 shows the comparison of Wi-Fi RSS and magnetic field when a pedestrian walks along a corridor in two opposite directions. The magnetic field shows good consistence with the corridor in different directions, but on the contrary, the Wi-Fi RSS is changing.

(2) Location Differentiation Ability 

**Figure 3:**
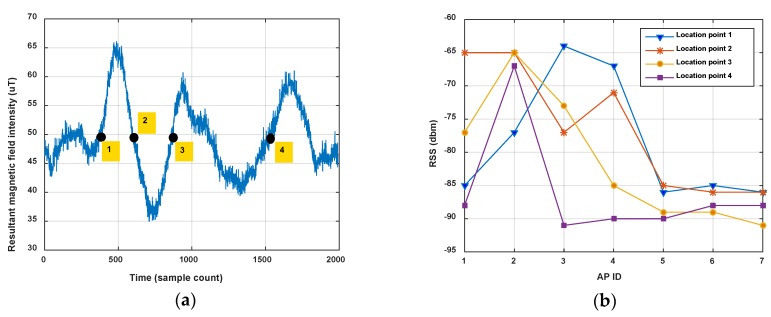
Comparison of the ability of location differentiation between magnetic field and Wi-Fi RSS: (**a**) Sequence of resultant magnetic field intensity in a corridor, at location points 1, 2, 3 and 4, where the intensities are same; (**b**) Difference RSS vectors at location points 1, 2, 3 and 4.

The measured magnetic field is the 3-axis magnetic intensity, and the intensity on each axis is dependent on the pose of the smartphone, so the resultant intensity is usually used to indicate the magnetic fingerprint. Therefore, for a single location point the magnetic fingerprint is a scalar, but the Wi-Fi fingerprint is a RSS vector whose dimensions will increase with the number of ambient APs. As shown in Figure 3a, the magnetic intensity is the same in some location points of one corridor (the resultant magnetic field intensities are all 50 μT at location points 1, 2, 3 and 4), but the Wi-Fi RSS vectors (seven APs are detected in the corridor) ate these location points are different, as shown in Figure 3b.

**Figure 4:**
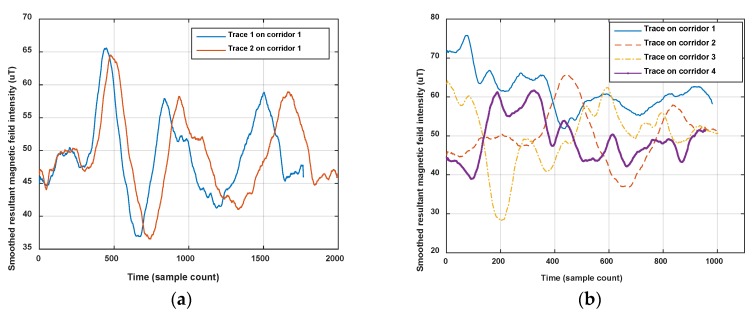
Comparison of magnetism sequences measured in the same corridor and different corridors: (**a**) Smoothed magnetism sequences generated in the same corridor; (**b**) Smoothed magnetism sequences generated in different corridors.

On the other hand, the magnetic fingerprint performs better in differentiation among corridors (or roads in indoor parking) than Wi-Fi, even when measured in the opposite direction (shown in Figure 2). Figure 4 shows the comparison of magnetism sequences measured in the same corridor and different corridors. It is obvious that magnetism sequences show highly similar shapes in the same corridor and low similarity in different corridors, so it is useful for crowdsourcing data to be divided into different corridors or clustered into same one. This is the principle for our proposed method. Since the similar shape of magnetic sequences in same corridor is based on the location correlation of the magnetic field; when the magnetic field time sequences are obtained by crowdsourcing users with different walking speeds (most of the time, users’ walking speeds are different, and hard to estimate accurately using crowdsourcing data), magnetism sequences would show an uncertain zooming state compared with the true space scale between two sample points.

In summary, the Wi-Fi fingerprint has a better performance in single location differentiation while the magnetic field shows better stability and ability in corridor division when used in sequence. Thus, a geomagnetism-aided method is designed to construct the indoor Wi-Fi radio-map via smartphone crowdsourcing in this paper.

### 2.2. Problem Setting

In this paper, we only concentrate on the 2D indoor localization problem. The crowdsourcing data collected by smartphone users are utilized to construct a Wi-Fi radio map automatically. Crowdsourcing data mentioned here generally include acceleration, angular velocity, magnetic field, orientation and Wi-Fi RSS provided respectively by the accelerometer, gyroscope, magnetometer, electronic compass and Wi-Fi connector mounted on a user’s smartphone. These data are generated and uploaded by crowdsourcing users when they are walking around the indoor environment, and meanwhile, recorded by the time identification stamp. Assuming that these data are continuous in time for each user, and we call them the moving traces of users, which are denoted as T. Each moving trace T is recorded as:

(2) T={Ori, Acc, Gyro, Mag, F, t}

Ori is the user’s moving orientation valued in [0°,360°) which is the reading from the electronic compass. However, due to magnetic field anomalies in indoor environments and the unconstrained smartphone poses of crowdsourcing users, it is essential to estimate the user’s heading using acceleration, angular velocity and magnetic field by some better algorithm rather than using readings directly from the electronic compass. The heading estimation method raised in [30] is a kind of solution for the orientation problem. Acc is the 3-axis acceleration, denoted as $\left(Acc_{x},Acc_{y},Acc_{z}\right)$. Gyro is the 3-axis angular velocity, denoted as $\left(Gyro_{x},Gyro_{y},Gyro_{z}\right)$. Mag is the 3-axis magnetic field intensity, denoted as $\left(Mag_{x},Mag_{y},Mag_{z}\right)$. F is the Wi-Fi fingerprint, include of AP Mac and RSS, denoted as $\left\{(Mac_{m},RSS_{m}),m=1,2,\ldots ,M\right\}$, here M is the total number of Wi-Fi APs scanned by the smartphone and t is the time identification.

**Figure 5:**
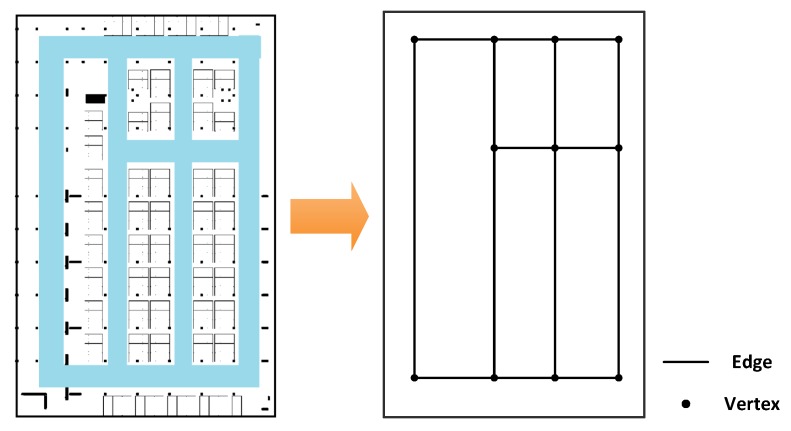
The indoor map is handled as a pathway graph in this paper: (**a**) a floor plan of an underground parking, the blue line is the pathway; (**b**) pathway graph of the left floor plan, the edges represent roads and the vertexes represent connections between roads.

Since indoor localization generally happens in corridors (or roads in indoor parking) which lead to rooms (or function sections) and entries of a floor (like stairs and elevators); in our method, the indoor map is handled as a pathway graph and indicated by topology. As shown in Figure 5, in a pathway graph of the indoor plan, the edges represent all the pathways that users can walk from one place to another; and the vertexes represent the turning corners and the endings of pathways. The indoor graph is denoted as Map in this paper, which is described as:

(3) Map={V, E}

V is a set of coordinates of all the vertexes in the graph, denoted as $\left\{(Coor_{m}),m=1,2,\ldots ,M\right\}$. In this paper, we use 2D coordinates to describe V, so, the vector V can also be denoted as $\left\{(x_{m},y_{m}),m=1,2,\ldots ,M\right\}$. Here M is the total number of vertexes. E is a matrix to represent the length of each edge between two vertexes, denoted as $\left\{(d_{p,q}),p=1,2,\ldots ,M,q=1,2,\ldots ,M\right\}$, here $d_{p,q}$ is Euclidean distance between *p*-th and *q*-th vertex.

In order to construct an indoor pathway graph by crowdsourcing user’s moving traces in our algorithm, the user’s trace will be broken down into some distinct road segments like the edges in a map. A road segment trace is a continuous trace with the turning connection or pathway ending only on its start or end points. The road segment R is denoted as:

(4) R={Rori, Ori, Acc, Gyro, Mag, F, t}

Rori is the mean value of Ori in the road segment trace, and it is a scalar identifying the displacement orientation of the road segment, valued in [0°,360°). And other elements in R are defined as same as T. The connection between two road segments is denoted as I, which is recorded as:

(5) $$I=\{R_{i},R_{j},Type,Angle\}$$

**Figure 6:**
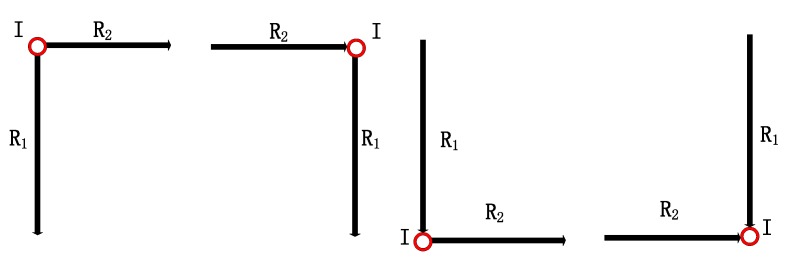
Four kinds of connection type defined in this paper. R represents a road segment, and I represents a connection.

It represents the connection between road segment $R_{i}$ and $R_{j}$. Type is the connection type including four kinds as shown in Figure 6, which is defined as:

(6) $$Type=\{\begin{array}{l} \begin{array}{cc} 1, & R_{i}\;\mathrm{start}\;\mathrm{connect}\;\mathrm{to}\;R_{j}\;\mathrm{start}\; \end{array}\\ \begin{array}{cc} 2, & R_{i}\;\mathrm{start}\;\mathrm{connect}\;\mathrm{to}\;R_{j}\;\mathrm{end} \end{array}\\ \begin{array}{cc} 3, & R_{i}\;\mathrm{end}\;\mathrm{connect}\;\mathrm{to}\;R_{j}\;\mathrm{start} \end{array}\\ \begin{array}{cc} 4, & R_{i}\;\mathrm{end}\;\mathrm{connect}\;\mathrm{to}\;R_{j}\;\mathrm{end} \end{array} \end{array}$$

**Figure 7:**
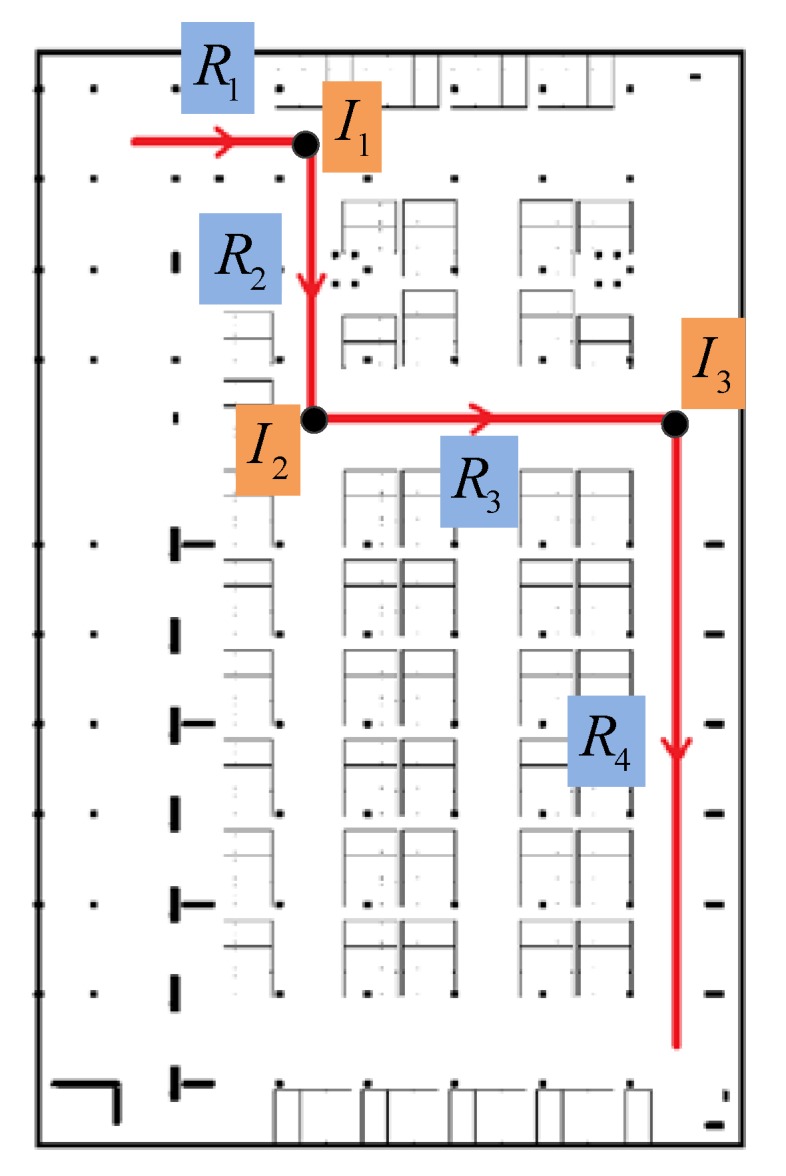
Example of connections between road segments. The red line is a user trace in an underground parking. R and I are respectively the road segments and connections in this trace.

Angle is the rotation angle between two road segments $R_{i}$ and $R_{j}$ in connection I, defined as the angle rotated from vector $R_{i}$ to vector $R_{j}$. The value of Angle is in (−180°,180°), negative for clockwise rotation and positive for anticlockwise rotation. Figure 7 shows an example of connection between road segments. It’s obvious that there are four road segments ($R_{1}$, $R_{2}$, $R_{3}$ and $R_{4}$) and three connections ($I_{1}$, $I_{2}$ and $I_{3}$) in this moving trace. The connections will be denoted respectively as $I_{1}=\{R_{1},R_{2},3,-90{}^{\circ}\}$, $I_{2}=\{R_{2},R_{3},3,90{}^{\circ}\}$ and $I_{3}=\{R_{3},R_{4},3,-90{}^{\circ}\}$.

Finally, it is expected that through the proposed method the topology of the indoor map (pathway graph) is acquired by crowdsourcing user traces, and Wi-Fi fingerprints are labeled on the map. The constructed Wi-Fi fingerprint database is represented as:

(7) $$\begin{array}{c} FD=\left\{\begin{array}{c} (x_{1},y_{1}),\;\;\;F_{1}\\ (x_{2},y_{2}),\;\;\;F_{2}\\ \ldots \\ (x_{N},y_{N}),\;\;\;F_{N} \end{array}\right\}\;\\ F_{n}=(RSS_{n}^{AP_{1}},RSS_{n}^{AP_{2}},\ldots ,RSS_{n}^{AP_{M}}) \end{array}$$

where N is the total number of RPs. M is the number of available Wi-Fi APs in the area.

### 2.3. Algorithm Overview

**Figure 8:**
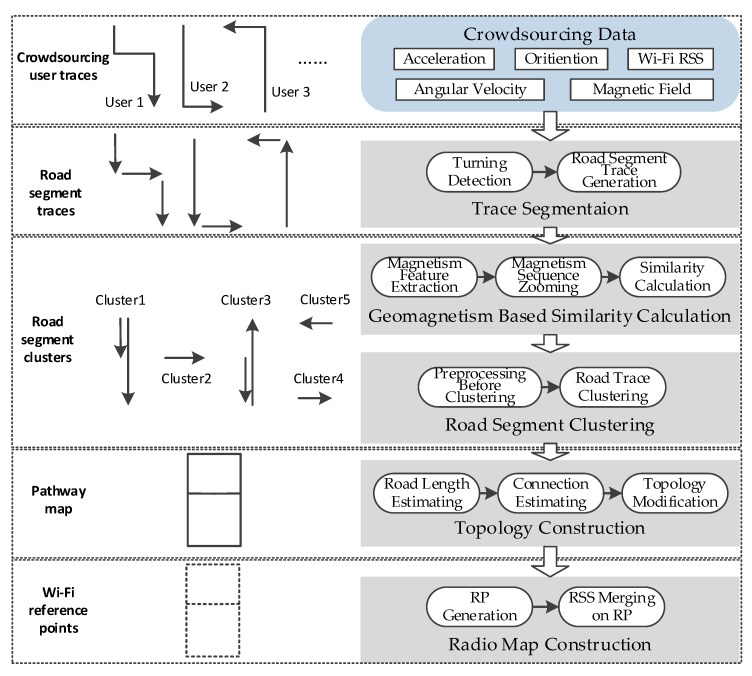
Overview of the geomagnetism-aided indoor Wi-Fi radio map construction method via smartphone crowdsourcing.

Through the discussion on the opportunities and challenges of smartphone-based indoor localization, a method of geomagnetism-aided indoor radio-map construction via smartphone crowdsourcing is proposed in this paper. In this method, the acceleration, angular velocity, orientation, Wi-Fi RSS and magnetic field from crowdsourcing data are utilized to realize the algorithm. The architecture of our method is shown in Figure 8, which includes five parts: trace segmentation, geomagnetism-based similarity calculation, road segment clustering, topology construction and final radio map construction.

First of all, the turning detection using angular velocity is implemented for the users traces, and the long traces will be segmented into some short ones which are generated in distinct corridors (or roads), called road segment traces.

Since there are usually more than one user trace generated in a corridor (road) in crowdsourcing data, we design a clustering method here to make these road segment traces from different users match together and cluster them into some distinct corridors. In order to realize clustering, a kind of matching method for road segment traces is designed by calculating the similarity of magnetism sequences of these, based on the stable shape of the magnetic field in one corridor (road). As discussed in Section 2.1, to deal with the uncertain zooming of magnetism sequences for different user traces, the magnetism features are extracted firstly, and then based on them one sequence is zoomed to a same distance scale as another one and consequently, the similarity of two road segment traces is calculated exactly.

Supported by the magnetism similarity, road segment traces are clustered to some collections with highly magnetic similarity. The clustering algorithm is designed based on the Density-Based Spatial Clustering of Application with Noise (DBSCAN) algorithm, using a kind of magnetism similarity neighborhood. In addition, a preprocessing step is implemented before clustering to make the magnetism sequences be generated based on the same orientation.

Furthermore, the length of road segments and connections between them are estimated, and then the topology map is constructed. To deal with the topology mistakes caused by inaccurate connection angles and road lengths, the topology modification is carried out to get a final pathway graph. Finally, the crowdsourcing Wi-Fi RSSs are carefully labeled by location coordinates, and merged together on the generated Wi-Fi RPs along the constructed pathway graph. Thus, the final Wi-Fi radio map (fingerprint database) is constructed on the floor.

## 3. Algorithm Details

### 3.1. Trace Segmentation

**Figure 9:**
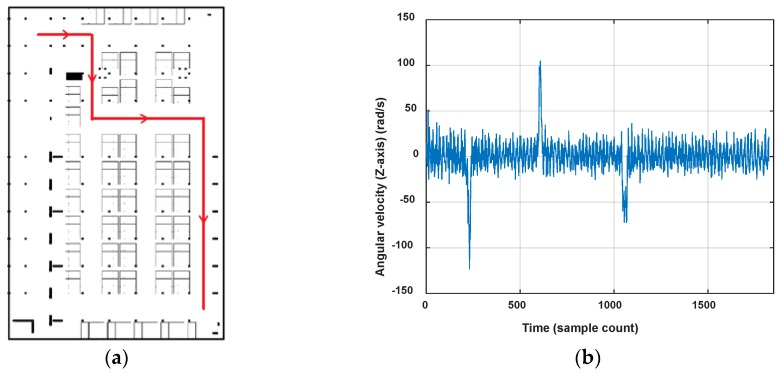
User’s walking trace and the angular velocities measured by a smartphone gyroscope: (**a**) walking trace in an underground parking with three turns; (**b**) angular velocities (vertical component) of the smartphone measured with the walking trace.

In this paper, only the 2D indoor localization problem is considered. Considering the features of the pathway graph, we segment a user’s trace through turn detection. The variation of orientations can obviously show the turning behavior of the user, but considering the heading errors caused by magnetic field anomalies in indoor environments and unconstrained smartphone poses, the reading changes of the gyroscope are used to detect user’s turning instead. Figure 9 shows a user’s walking trace and the vertical component of the angular velocity. When the pedestrian walks straight, the values of angular velocity oscillate up and down around zero. On the contrary, when the pedestrian turns left or right, the absolute value of angular velocities will rise to a peak and then decline to normal, corresponding with the turn start and finish. The change would be obvious for quickly turning and gentle for a slow turn. Moreover, it shows that the angular velocities will go negative for clockwise turning and positive for anticlockwise turning.

As discussed above, in this paper, we use angular velocity readings from the gyroscope to detect turns, calculate turning angles and finally segment user’s traces. Before turn detection, the vertical component of the angular velocity is calculated by gravitational acceleration and coordinate transformation. For dealing with the measurement noise, the angular velocity will be smoothed using a moving average filter before turn detection. Three rules are made as follows to realize turning detection and turning angle estimation:

- The peaks of angular velocities are detected when the following conditions are satisfied. Here $Th_{Turn}$ is a positive constant. The position of each peak is recorded using the sample count *n*:
  (8) $$P=\{\begin{array}{l} \begin{array}{cc} n, & \begin{array}{l} Gyro(n)>Th_{Turn},Gyro(n)>Gyro(n-1),Gyro(n)>Gyro(n+1)\\ or\;Gyro(n)<-Th_{Turn},Gyro(n)<Gyro(n-1),Gyro(n)<Gyro(n+1) \end{array} \end{array}\\ \begin{array}{cc} null, & else \end{array} \end{array}$$
- The start of turn $n_{1}$ is settled as the first point ascending from zero to the peak, and the end of turn $n_{2}$ is settled as the last declining point from the peak to zero.
- The user trace is segmented by detected peaks to some road segments, denoted as $\{R_{i}\},i=1,2,\ldots ,\mathrm{num}(P)+1$. num(P) is the number of peaks detected. The orientation of each road segment Rori is the mean value of data Ori in this road; and the rotation angle of the turning Angle is the integration from $Gyro(n_{1})$ to $Gyro(n_{2})$, shown in the following equations:
  (9) $$Rori_{Ri}=\bar{\sum_{n=P(i-1)}^{P(i)}Ori(n)}$$
  (10) $$Angle=\int_{n_{1}}^{n_{2}}Gyro\;dn$$

**Figure 10:**
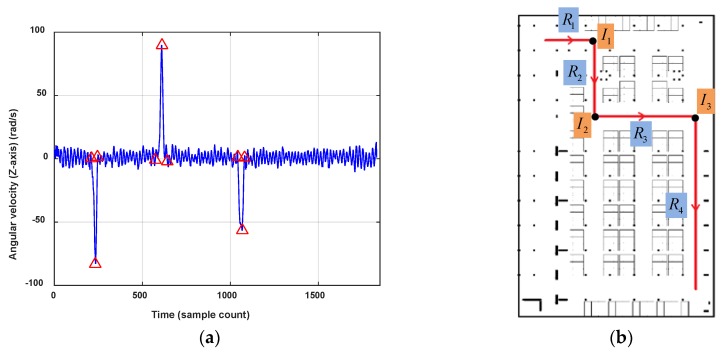
Turn detection results using angular velocity: (**a**) vertical component of the angular velocities (smoothed) of the test trace and the detected start point, peak and end point of each turn; (**b**) four segmented road traces and the three connections between them.

Figure 10 shows the turn detection result when setting $Th_{Turn}=40{}^{\circ}$. There are three turns detected in this trace T. Then, we segment the user trace T into four road segment traces $R_{1}$, $R_{2}$, $R_{3}$ and $R_{4}$, and three connections, which are denoted respectively by $I_{1}=\{R_{1},R_{2},3,-79.2{}^{\circ}\}$, $I_{2}=\{R_{2},R_{3},3,95.5{}^{\circ}\}$ and $I_{3}=\{R_{3},R_{4},3,-80.4{}^{\circ}\}$. To deal with smartphone pose changes (false detection) and gentle turns (undetected) of the user, the turn detection result would be checked again by the user headings. If the offset of Rori between two separate road segment traces are close to the detected turning angle, it is proved that there is a real turn. If the orientation change happens inside one road segment trace, the truing detection algorithm will be re-implement using an angular velocity with a lower $Th_{Turn}$. Of course, the erroneous road segments can also be eliminated by failing magnetic field matching in the following process.

### 3.2. Geomagnetism-Based Similarity Calculation

As discussed in Section 2.1, the geomagnetism sequence in the road segment obtained in the above process will be utilized to calculate the similarity between two road segment traces in our proposed method. To deal with the different scales of sequences, [6] used a Dynamic Time Warping (DTW) algorithm for sequence similarity computation, but DTW algorithms need a large amount of calculation, so in this section, we propose a novel method for similarity calculation using geomagnetism features. The details of geomagnetism-based similarity calculation will be described below, consisting of magnetism feature extraction, sequence zooming based on matched feature points, and final similarity calculation.

#### 3.2.1. Feature Extraction

The geomagnetism measurements from a smartphone are 3-axis magnetism intensities (one reading for each axis). Considering the uncertain pose of smartphones, which causes measuring coordinate system changes, before we extract magnetism features from a data sequence, the vector module of the magnetic field is calculated firstly as the resultant magnetism intensity. The resultant magnetism intensity is acquired by the following equation, and only related to the position of the smartphone:

(11) $$Mag=\sqrt{Mag_{x}^{2}+Mag_{y}^{2}+Mag_{z}^{2}}$$

We define peaks and troughs from the sequence of resultant magnetism intensities **Mag** which satisfy the settled constraints as the magnetism features of the user trace. To remove the noise in magnetism sequence and extract its main shape, we smooth the magnetism vector using a moving average filter before feature extraction. The magnetism features are identified by the following criteria:

- All the peaks and troughs are detected from sequence **Mag** by the following equation, and **MP** and **MT** are respectively peak candidates and trough candidates:
  (12) $$\{\begin{array}{cc} \begin{array}{c} MP=n,\\ MT=n, \end{array} & \begin{array}{l} Mag(n)>Mag(n-1)\;\mathrm{and}\;Mag(n)>Mag(n+1)\\ Mag(n)<Mag(n-1)\;\mathrm{and}\;Mag(n)<Mag(n+1) \end{array} \end{array}$$
- The candidates (both peaks and troughs together), whose magnetism intensity difference with one of the two interfacing candidates is below the defined threshold thMagDiff, are removed from candidates.
- Peaks and troughs that satisfy the above constraint are the final magnetism features which are marked by *P* and *T*, respectively. At the same time, peaks and troughs would be ensured to appear alternately.

**Figure 11:**
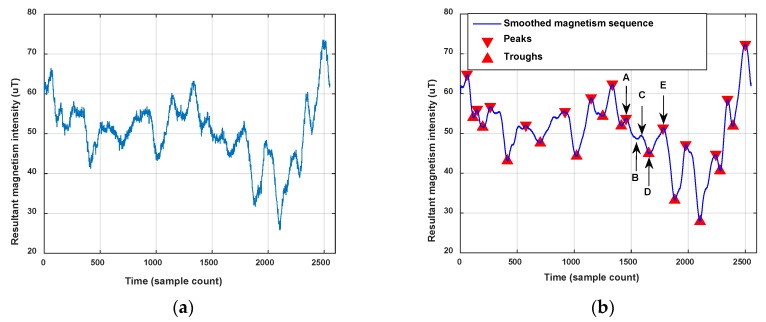
Example for magnetism feature extraction: (**a**) sequence of resultant magnetism intensity generated with a user trace; (**b**) feature extraction result.

Figure 11 shows an example of a magnetism feature extraction result. Points A, C and E marked in the figure are detected peaks, and B and D are detected troughs. When we set thMagDiff as 1 μT, the difference between B and A exceeds thMagDiff, but the difference between B and C doesn’t exceed thMagDiff, so trough point B is not a feature point we need. On the contrary, peak D is a feature point, as the difference between D and A as well as D and E both exceed the thMagDiff. Consequently, as shown in Figure 11, the trough B and the peak C would be removed since they don’t satisfy the constraint, but the trough D and the peaks A and E are the extracted magnetism features, so though the abovementioned method, all the peaks and troughs are extracted as the magnetism features that can describe the fluctuation of the resultant magnetism intensity in the user’s trace.

#### 3.2.2. Sequence Zooming

In this part, we propose a method to zoom the distance scale of one magnetism sequence to another through magnetism features in order to match them together. It includes four key steps:

- *Step 1*: First of all, we estimate the movement distance from the start point of the user trace to each magnetic field sampling time using an empirical stride length. Then two pairs of magnetism features from the two sequences are selected randomly as the initial assumed matching feature points, if the ratio of two estimated distances between features in each sequence is within a reasonable range of pedestrian stride length.
- *Step 2*: Secondly, using the distance ratio calculated by the initial matching feature pairs, the remaining part of the second sequence is zoomed to the same stride length as the first one.
- *Step 3*: Thirdly, to deal with the common case of uneven walking speed in one trace, the rest of the feature points from two sequences are matched together based on the initial matched features.
- *Step 4*: Finally, the number of matched feature points is counted, and if the proportion of matched features is greater than the settled threshold, one time of Sequence Zooming is finished.

The details of the above steps will be respectively depicted in the following paragraphs.

(1) Initial Feature Matching

We use detected steps and stride length to estimate the trace length. The acceleration is utilized for step detection. The algorithm for step detection is the same as the Feature Extraction algorithm presented in 3.2.1. The detected peaks of resultant acceleration which satisfy the settled constraint are user steps, and the amount of steps is marked as $N_{step}$. Other algorithms can also be used to realize step detection, like the one described in [31]. Stride length $L_{step}$ is set as an empirical value like 0.6 m, so the length of the user trace is estimated by:

(13) $$L=N_{step}\cdot L_{step}$$

The trace length L do not need to be exact, as it is only used to provide a rough distance between each magnetism feature, and the similarity of the queues of magnetism features is the main criterion to estimate whether two traces are generated on the same road segment. The procedures of features alignment and similarity calculation will be proposed in the following context.

When we get the length of the trace, the distances from start point of the trace to each magnetism sampling time, denoted as $Dis=\left\{Dis_{1},Dis_{2},Dis_{3},\ldots ,Dis_{M}\right\}$, can be estimated by:

(14) $$Dis_{i}=L\cdot \frac{i}{M},\;i=1,2,3,\ldots ,M$$

Here i is the *i*-th count of magnetic field readings, M is the total number of the field data on a road segment trace. We use a linear model here to estimate distance each time, for it is hard to get the exact walking speed of users, and the linear moving model will simplify the procedures to acquire a rough distance sequence. In case of the uneven walking speed in one trace, the magnetism features alignment will be implemented in Step 3.

Considering two road segment data of different users, denoted by R1 and R2. The magnetism sequences of them are $Mag^{1}$ and $Mag^{2}$, denoted as $Mag^{1}=\{Mag_{1}^{1},\;Mag_{2}^{1},\;Mag_{3}^{1}\ldots Mag_{M}^{1}\}$ and $Mag^{2}=\{Mag_{1}^{2},\;Mag_{2}^{2},\;Mag_{3}^{2}\ldots Mag_{N}^{2}\}$. The data length of each sequences are respectively M and N. Though the above mentioned method, the distance sequences corresponding to $Mag^{1}$ and $Mag^{2}$ are obtained, denoted as $Dis^{1}=\left\{Dis_{1}^{1},\;Dis_{2}^{1},\;Dis_{3}^{1},\;\ldots \;,\;Dis_{M}^{1}\right\}$ and $Dis^{2}=\left\{Dis_{1}^{2},\;Dis_{2}^{2},\;Dis_{3}^{2},\;\ldots \;,\;Dis_{N}^{2}\right\}$.

Then the magnetism features are extracted from $Mag^{1}$ and $Mag^{2}$, recorded by $P^{1}$, $T^{1}$, $P^{2}$ and $T^{2}$:

(15) $$\begin{array}{l} P^{1}=\{P_{1}^{1},\;P_{2}^{1},\;P_{3}^{1},\ldots ,P_{M_{P}}^{1}\}\\ T^{1}=\{T_{1}^{1},\;T_{2}^{1},\;T_{3}^{1},\ldots ,T_{M_{T}}^{1}\}\\ P^{2}=\{P_{1}^{2},\;P_{2}^{2},\;P_{3}^{2},\ldots ,P_{N_{P}}^{2}\}\\ T^{2}=\{T_{1}^{2},\;T_{2}^{2},\;T_{3}^{2},\ldots ,T_{N_{T}}^{2}\} \end{array}$$

$M_{P}$ and $M_{T}$ are respectively the total numbers of peaks and troughs in $Mag^{1}$. Similarly, $N_{P}$ and $N_{T}$ are respectively the total numbers of peaks and troughs in $Mag^{2}$.

At the beginning of Sequence Zooming, we pick up two couples of magnetism features respectively from $Mag^{1}$ and $Mag^{2}$ randomly. They are recorded by $FP_{1}^{1}$, $FP_{2}^{1}$ (a feature point pair from $Mag^{1}$) and $FP_{1}^{2}$, $FP_{2}^{2}$ (a feature point pair from $Mag^{2}$). The following constraint should be satisfied in selection of feature point pairs:

(16) $$\begin{array}{l} (FP_{1}^{1},FP_{2}^{1})=(P_{a}^{1},P_{b}^{1})\;\mathrm{or}\;(T_{a}^{1},T_{b}^{1})\;\mathrm{or}\;(P_{a}^{1},T_{b}^{1})\;\mathrm{or}\;(T_{a}^{1},P_{b}^{1}),\;a<b\\ (FP_{1}^{2},FP_{2}^{2})=(P_{c}^{2},P_{d}^{2})\;\mathrm{or}\;(T_{c}^{2},T_{d}^{2})\;\mathrm{or}\;(P_{c}^{2},T_{d}^{2})\;\mathrm{or}\;(T_{c}^{2},P_{d}^{2}),\;c<d \end{array}$$

The above equation means that the feature point pairs from $Mag^{1}$ and $Mag^{2}$ should have the same pattern and same order in their queues. For example, when we choose $\left(P_{a}^{1},T_{b}^{1}\right)$ as feature point couple in $Mag^{1}$, we should use $\left(P_{c}^{2},T_{d}^{2}\right)$ in $Mag^{2}$ to match with them. The subscripts *a*, *b*, *c* and *d* should meet the conditions a<b and c<d.

If $FP_{1}^{1}$ and $FP_{1}^{2}$ are generated at a same location point A, and at the same time, $FP_{2}^{1}$ and $FP_{2}^{2}$ are at a same location point B, the trace distances between $FP_{1}^{1}$ and $FP_{2}^{1}$, as well as $FP_{1}^{2}$ and $FP_{2}^{2}$ would be equal, assuming that the walking paths on the road segment are unique for users, so we can obtain the following equality:

(17) $$N_{step}^{1}\cdot L_{step}^{1}=N_{step}^{2}\cdot L_{step}^{2}=L_{AB}$$

in which, $N_{step}^{1}$ and $N_{step}^{2}$ are respectively the number of detected steps from location A to B on road segment traces R1 and R2. $L_{step}^{1}$ and $L_{step}^{2}$ are the real stride length of users on R1 and R2. $L_{AB}$ is the actual trace length between location A and B. Stride length $L_{step}$ is the empirical value that we use to estimate the distances from the start point to each magnetic field sampling point, so it can also be obtained that:

(18) $$N_{step}^{1}\cdot L_{step}=Dis^{1}(FP_{2}^{1})-Dis^{1}(FP_{1}^{1})$$

(19) $$N_{step}^{2}\cdot L_{step}=Dis^{2}(FP_{2}^{2})-Dis^{2}(FP_{1}^{2})$$

Then the stride length ratio is reckoned as:

(20) $$ratio_{L}=\frac{L_{step}^{2}}{L_{step}^{1}}=\frac{N_{step}^{1}}{N_{step}^{2}}=\frac{N_{step}^{1}\cdot L_{step}}{N_{step}^{2}\cdot L_{step}}=\frac{Dis^{1}(FP_{2}^{1})-Dis^{1}(FP_{1}^{1})}{Dis^{2}(FP_{2}^{2})-Dis^{2}(FP_{1}^{2})}$$

The normal range of pedestrian stride lengths is about 0.4 m to 0.8 m, so the stride length ratio $ratio_{L}$ should be on the range of [0.5, 2]. When the calculated $ratio_{L}$ is in this range, the feature points ($FP_{1}^{1}$,$FP_{1}^{2}$) and ($FP_{2}^{1}$,$FP_{2}^{2}$) can be assumed as the initial matched feature points of R1 and R2. On the contrary, if $ratio_{L}$ is out of this range, this procedure will be implemented again for another pair of feature points, until the initial matched points are found.

(2) Rough Zooming

Based on the initial matched feature points ($FP_{1}^{1}$,$FP_{1}^{2}$), the data on $Dis^{2}$ will be zoomed using $ratio_{L}$, and $Dis^{2}(FP_{1}^{2})$ will be translated to $Dis^{1}(FP_{1}^{1})\;$. The new distance sequence is calculated by the following equation:

(21) $$Dis^{2}{}_{Z}(n)=(Dis^{2}(n)-Dis^{2}(FP_{1}^{2}))\cdot ratio_{L}+Dis^{1}(FP_{1}^{1})\;$$

here n=1,2,3,…,N. Then we get a new distance sequence $Dis^{2}{}_{Z}$ for R2. $Dis^{2}{}_{Z}$ may have some negative data, for its start point would not be the same as for R1.

After rough zooming in the above procedure, the magnetism feature points on R2 are basically matched together with those on R1 if the initial matching points ($FP_{1}^{1}$,$FP_{1}^{2}$) are correct. Because of the rough zooming is an even zooming only based on the scalar $ratio_{L}$, if users’ walking speeds are uneven, which is a very common situation, the feature points will not match well only by rough zooming. The following step is designed to deal with this issue.

(3) Features Alignment

In this step, the feature points in R2 are aligned based on the current initial matched feature points (($FP_{1}^{1}$,$FP_{1}^{2}$) and ($FP_{2}^{1}$,$FP_{2}^{2}$)) and matched with other feature points in R1 using the principle of proximity. Therefore, when the initial matched feature points are really sampled on the same location point, the Features Alignment would make other feature points on R2 and R1 match exactly, so that the magnetism similarity of R1 and R2 can be calculated accurately. When the initial assumed matching feature points are not matched correctly, this procedure will also be carried out and the final matching result will be judged by the result of Similarity Calculation in the following step.

We denote the assemblage of aligned feature points as AF. Based on the initial matched feature points, the initial of the assemblage AF is shown in the following:

(22) $$AF=\{AF_{1},AF_{2}\},AF_{1}=(FP_{1}^{1},FP_{1}^{2}),AF_{2}=(FP_{2}^{1},FP_{2}^{2})$$

For the peak $P_{i}^{2}$, the candidate matching area in R1 is settled at first, which is indicated by (IndexL,IndexR). The edge of matching area is the feature points of R1 in AF, whose matched feature points in R2 are the closest ones respectively to left and right side of $P_{i}^{2}$. This can be expressed as:

(23) $$\{\begin{array}{c} IndexL=FP^{1}\underset{k}{(\arg\min}\{P_{i}^{2}-FP_{k}^{2}\}),FP_{k}^{2}<P_{i}^{2}\\ IndexR=FP^{1}\underset{k}{(\arg\min}\{FP_{k}^{2}-P_{i}^{2}\}),FP_{k}^{2}>P_{i}^{2} \end{array}$$

When $P_{i}^{2}$ only has one side neighbor with aligned feature points, then the matching area will be settled as (1,IndexR) or (IndexL,M). M is the data length of $Mag^{1}$.

Then all the peaks of R1 in the range of (IndexL,IndexR) are traversed to find the matching point of $P_{i}^{2}$. The matching point $match(P_{i}^{2})$ would satisfy two requirements, including:

- The difference between $Dis^{2}{}_{Z}(P_{i}^{2})$ and $Dis^{1}(P_{k}^{1})$ is minimum compared with other candidate peaks. For the $Dis^{2}{}_{Z}$ have been accorded with $Dis^{1}$, the difference between them indicates the probable location distance between $P_{i}^{2}$ and $P_{k}^{1}$, and the closest pair may have the biggest probability to be sampled on a same location.
- In case of some feature points are undetected, and R1 and R2 have different trace lengths, we set a distance threshold thAlign to restrict the difference of distance between matching points. The thAlign in this paper is settled as an empirical value.

The matching qualification is expressed by the following equation:

(24) $$match(P_{i}^{2})=P_{k}^{1},\{\begin{array}{l} \underset{k}{k=\arg\min}\{\left|Dis^{2}{}_{Z}(P_{i}^{2})-Dis^{1}(P_{k}^{1})\right|\}\\ \left|Dis^{2}{}_{Z}(P_{i}^{2})-Dis^{1}(P_{k}^{1})\right|<thAlign\\ P_{k}^{1}\in [IndexL,IndexR] \end{array}$$

The matched peak couples would be added into AF as a new element $AF_{j}=(FP_{j}^{1},FP_{j}^{2})=(P_{k}^{1},P_{i}^{2})$, and further used for matching area choosing of the new $P_{i}^{2}$ prepared for alignment.

For the troughs $T_{i}^{2}$, the same method is implemented to find the matching troughs $T_{k}^{1}$ in R1. At the same time, the matched trough couples will also be added into AF as a new element, recorded by $AF_{j}=(FP_{j}^{1},FP_{j}^{2})=(T_{k}^{1},T_{i}^{2})$.

For the matched feature couples should be apparent in same order both in R1 and R2, and peaks and troughs should appear alternately, the element in AF (all the matched peaks and troughs) will be resolved by the data order of feature points in $Mag^{1}$ (or feature points in $Mag^{2}$). Finally, we obtained all the matched feature couples in AF:

(25) $$AF=\{AF_{j}\},\;AF_{j}=(FP_{j}^{1},FP_{j}^{2}),j=1,2,\ldots ,J$$

J is the total number of matched feature couples between R1 and R2.

Then, based on the matched feature pairs in AF, the $Dis^{2}{}_{Z}$ would be updated using the following equation:

(26) $$Dis^{2}{}_{A}(n)=\{\begin{array}{ll} (Dis^{2}{}_{Z}(n)-Dis^{2}{}_{Z}(FP_{j}^{2}))\cdot \frac{Dis^{1}(FP_{j+1}^{1})\;-Dis^{1}(FP_{j}^{1})}{Dis^{2}{}_{Z}(FP_{j+1}^{2})\;-Dis^{2}{}_{Z}(FP_{j}^{2})}+Dis^{1}(FP_{j}^{1}), & n\in [FP_{j}^{2},FP_{j+1}^{2}],j\in [1,J-1]\\ Dis^{2}{}_{Z}(n)-Dis^{2}{}_{Z}(FP_{1}^{2})+Dis^{1}(FP_{1}^{1}), & n\in [1,FP_{1}^{2})\\ Dis^{2}{}_{Z}(n)-Dis^{2}{}_{Z}(FP_{J}^{2})+Dis^{1}(FP_{J}^{1}), & n\in (FP_{J}^{2},N] \end{array}$$

It means that all the matched feature points $FP_{j}^{2}$ in R2 will be aligned to the same distance of $FP_{j}^{1}$ in R1 and the distance of other sample points will be zoomed using the local ratio, estimated by the closest two feature point couples. In the start or end part of the R2, only translation is implemented to the $Dis^{2}{}_{Z}(n)$, since the zooming ratio cannot be estimated only by one pair of matched feature point. Finally, we get the updated distance sequence $Dis^{2}{}_{A}$ for R2.

(4) Matched Features Amount Judgment

In this step we count the amount of matched feature couples in AF, and calculate the matching proportion $P_{FP}$ using the following equation:

(27) $$P_{FP}=\frac{J}{\min(M_{P}+M_{T},N_{P}+N_{T})}$$

$M_{P}$ and $M_{T}$ are respectively total numbers of peaks and troughs in $Mag^{1}$. $N_{P}$ and $N_{T}$ are respectively the total numbers of peaks and troughs in $Mag^{2}$. J is the total number of matched feature pairs between $Mag^{1}$ and $Mag^{2}$. When $P_{FP}$ is greater than the settled threshold thFP, one time of Sequence Zooming is finished.

#### 3.2.3. Similarity Calculation

Through the method of Sequence Zooming provided in the above section, the distance scales of $Mag^{1}$ and $Mag^{2}$ are basically uniform. If the feature points are matched correctly, the similarity between $Mag^{1}$ and $Mag^{2}$ can be calculated accurately. In this paper, the correlation coefficient of two magnetism sequences is identified as the similarity of two road segment traces R1 and R2.

Before correlation coefficient calculation, the data length of $Mag^{1}$ and $Mag^{2}$ would be made to be equal using original magnetism data and zoomed distance data, based on linear interpolation. The equation to calculate the new magnetism sequence $Mag^{2}{}_{new}$, which has the same data length with $Mag^{1}$, is shown in the following:

(28) $$Mag^{2}{}_{new}(m)=\{\begin{array}{ll} Mag^{2}(n+1)-\frac{Dis^{2}{}_{A}(n+1)-Dis^{1}(m)}{Dis^{2}{}_{A}(n+1)-Dis^{2}{}_{A}(n)}\cdot (Mag^{2}(n+1)-Mag^{2}(n)), & \begin{array}{l} Dis^{1}(m)\in [Dis^{2}{}_{A}(n),Dis^{2}{}_{A}(n+1)],\\ n\in [1,N-1] \end{array}\\ \mathrm{Null} & \begin{array}{l} Dis^{1}(m)<Dis^{2}{}_{A}(1),\\ \mathrm{or}\;Dis^{1}(m)>Dis^{2}{}_{A}(N) \end{array} \end{array}\;$$

Symbol “Null” in the equation means that it is a null element in sequence $Mag^{2}{}_{new}$, and there isn’t a valid mathematical result in this element.

Then we find the index of valid element in $Mag^{2}{}_{new}$, and calculate the correlation coefficient between $Mag^{1}$ and $Mag^{2}{}_{new}$. The indexes of the start and end point of valid element are respectively denoted as $m_{start}$ and $m_{end}$. cc is the correlation coefficient between $Mag^{1}$ and $Mag^{2}{}_{new}$. They are obtained by:

(29) $$\begin{array}{c} m_{start}=\min(m|Mag^{2}{}_{new}(m)\neq \mathrm{Null})\\ m_{end}=\max(m|Mag^{2}{}_{new}(m)\neq \mathrm{Null}) \end{array}$$

(30) $$cc=\frac{\sum_{m=m_{start}}^{m_{end}}\left(Mag^{1}(m)-\bar{Mag^{1}})(Mag^{2}{}_{new}(m)-\bar{Mag^{2}{}_{new}}\right)}{\sqrt{\sum_{m=m_{start}}^{m_{end}}{\left(Mag^{1}(m)-\bar{Mag^{1}}\right)}^{2}\cdot \sum_{m=m_{start}}^{m_{end}}{\left(Mag^{2}{}_{new}(m)-\bar{Mag^{2}{}_{new}}\right)}^{2}}}$$

For each time of Sequence Zooming, the correlation coefficient cc would be calculated, until all the probable initial matched features in R1 and R2 are tested. Afterwards, the maximum of all the calculated cc between R1 and R2 is selected as the road segment similarity between R1 and R2, denoted as RS(R1,R2). Correspondingly, the final zooming ratio of R2 based on R1 (denoted as $Ratio(R_{2},R_{1})$) is calculated and given by:

(31) $$Ratio(R_{2},R_{1})=\frac{Dis^{2}{}_{A}(N)-Dis^{2}{}_{A}(1)}{Dis^{2}(N)}$$

The matching position between R1 and R2 is settled as $\left[(m_{start},n_{start}),(m_{end},n_{end})\right]$. $m_{start}$ and $m_{end}$ is obtained by Equation (29). $n_{start}$ and $n_{end}$ are ascertained by:

(32) $$\begin{array}{c} n_{start}=(n|Dis^{2}{}_{A}(n)=Dis^{1}(m_{start}))\\ n_{end}=(n|Dis^{2}{}_{A}(n)=Dis^{1}(m_{end})) \end{array}$$

#### 3.2.4. Similarity Calculation Result

**Figure 12:**
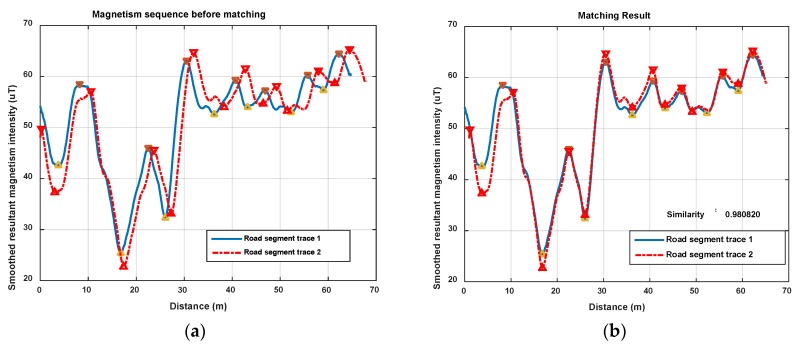
Magnetism matching and similarity calculation results for two traces generated on the same road segment: (**a**) smoothed magnetism sequences and detected features marked by the initial estimated distance; (**b**) magnetism sequences after feature matching and sequence zooming, and the their calculated similarity.

**Figure 13:**
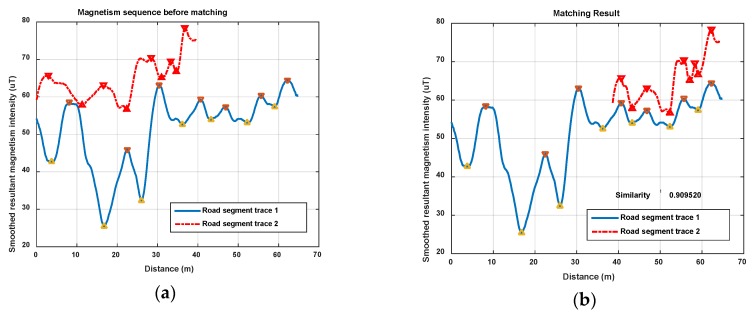
Magnetism matching and similarity calculation results when trace 2 is partly generated on the same road segment that trace 1 was generated on: (**a**) smoothed magnetism sequences and detected features marked by the initial estimated distance; (**b**) magnetism sequences after feature matching and sequence zooming, and the calculated similarity between them.

**Figure 14:**
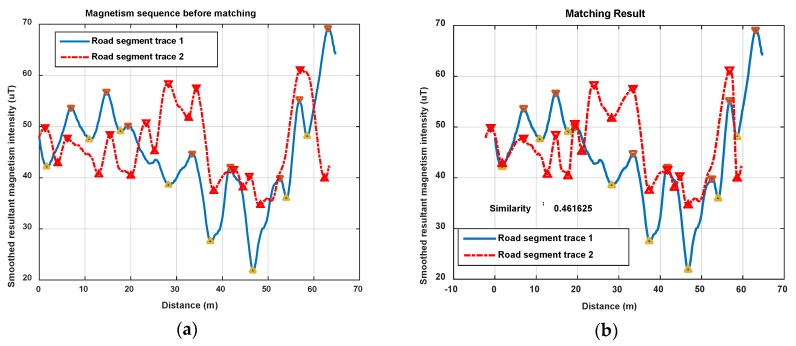
Magnetism matching and similarity calculation results for two traces generated on different road segments: (**a**) smoothed magnetism sequences and detected features marked by the initial estimated distance; (**b**) magnetism sequences after feature matching and sequence zooming, and the calculated similarity between them.

In this section, we show magnetism matching and similarity calculation results for some typical situations, including two traces generated on the same road segment and on different road segments. These results are shown in Figure 12, Figure 13 and Figure 14.

Figure 12 shows the magnetism matching and similarity calculation result for two traces generated on a same road segment. Figure 12a shows the smoothed magnetism sequences from road segment trace 1 and road segment trace 2, in which the distances from start point to each magnetic field sampling point are estimated by detected step and empirical stride length ($L_{step}=0.6\;m$). We can see that the magnetism sequences of the two traces have highly similar shape and different distance scales. Figure 12b shows the features matching result and similarity (correlation coefficient) of these two magnetism sequences. The distance threshold used in features alignment is settled as thAlign=2 m, and the minimum threshold of the proportion between matched feature couples in AF and the total number of features is settled as thFP=70%. It shows that through features matching and sequence zooming, the matched features are translated to a same distance point, and two magnetism sequences are overlapped to the highest extent. The calculated correlation coefficient (valued in [−1,1]) is 0.98, which shows a high similarity between this two traces, and the result agrees with the real situation.

Figure 13 and Figure 14 show another two situations using the same parameters as those in Figure 12. In Figure 13, trace 2 is partly generated on the road segment that trace 1 was generated on. Through feature matching and sequence zooming, trace 2 is matched closely to the right part of trace 1, and the correlation coefficient (=0.91) shows a high similarity between them. Furthermore, it also shows that the intensity offset between the magnetism sequences of these two traces didn’t impact the similarity calculation result, since the correlation is mainly dependent on the shape of the two sequences. In Figure 14, traces on different road segments are processed using our algorithm, some peaks and troughs are matched together, but the similarity is at a low level of 0.46.

As the results above show, the proposed geomagnetism-based similarity calculation algorithm is proved to have a good performance for judging the similarity between two road segment traces.

### 3.3. Road Segment Clustering

#### 3.3.1. Preprocessing Before Clustering

For one road segment, there are two possible walking directions for users, identified as the positive and the opposite direction. Due to this fact, a preprocessing step is implemented for the users’ road segment traces obtained by trace segmentation (shown in Section 3.1) before clustering to make the magnetism sequence be generated on a generally similar direction. Consequently the correlation coefficient between two magnetism sequences can be calculated correctly.

We use Rori in the user’s road segment trace *R* for walking direction judgment of two road segment traces (in the same direction or opposite direction). Rori is the mean value of all the Ori data in *R* (the details can be found in Section 3.1).

For two user’s road segment traces R1 and R2, the road orientations are denoted as $Rori_{1}$ and $Rori_{2}$. The difference between $Rori_{1}$ and $Rori_{2}$ is denoted as ΔRori. Considering the uniqueness of ΔRori, we use value between 0° and 180° to indicate it, and the value of ΔRori can be calculated by:

(33) $$\Delta Rori=\{\begin{array}{ll} \left|Rori_{1}-Rori_{2}\right|, & \left|Rori_{1}-Rori_{2}\right|\in [0{}^{\circ},180{}^{\circ}]\\ 360{}^{\circ}-\left|Rori_{1}-Rori_{2}\right|, & \left|Rori_{1}-Rori_{2}\right|\in (180{}^{\circ},360{}^{\circ}) \end{array}$$

**Figure 15:**
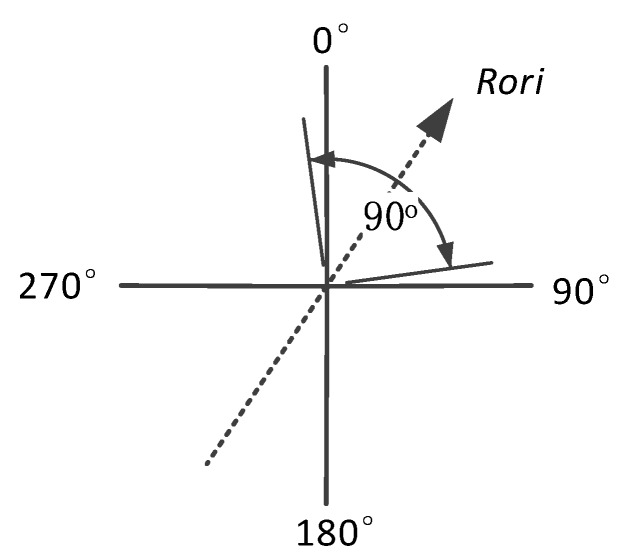
Coordinate system for road orientation and the uncertainty (±45°) of Rori shown in it.

Considering the orientation offset between Rori and the real orientation, we set ±45° as the uncertainty of road orientation Rori, as shown in Figure 15, so the true orientation of the road segment can be indicated by:

(34) $$Rori^{true}=Rori\pm 45{}^{\circ}$$

Here $Rori^{true}$ is the true orientation and Rori is the measured value. Thus, the difference between measured orientations ($Rori_{1}$ and $Rori_{2}$) can also be indicated by:

(35) $$\Delta Rori=\left|Rori_{1}-Rori_{2}\right|=\left|\left(Rori_{1}^{true}\pm 45{}^{\circ})-(Rori_{2}^{true}\pm 45{}^{\circ}\right)\right|$$

If R1 and R2 are generated on the same walking direction ($Rori_{1}^{true}=Rori_{2}^{true}$), the orientation difference ΔRori would fall in [0°,90°]. If R1 and R2 are generated on the opposite walking direction ($Rori_{1}^{true}=Rori_{2}^{true}+180{}^{\circ}$), the orientation difference ΔRori would be in [90°,180°].

As discussed above, we calculate the orientation difference ΔRori between R1 and R2, and implement data processing shown as following:

- When ΔRori∈[0°,90°], it is estimated that R1 and R2 are generated in the same direction, and the similarity calculation will be implemented directly between $Mag^{1}$ and $Mag^{2}$ using the algorithm shown in Section 3.2. Here $Mag^{1}$ and $Mag^{2}$ are the resultant magnetism sequences from R1 and R2, respectively.
- When ΔRori∈[90°,180°], it is estimated that R1 and R2 are generated in the opposite direction, so before similarity calculation, sequence $Mag^{2}$ would be reversed firstly, denoted as $Mag^{2}{}^{\prime }$, and then the similarity with $Mag^{1}$ calculated.
- If ΔRori=90°, as shown in the above two bullets, the similarity would be calculated twice using both $Mag^{2}$ and $Mag^{2}{}^{\prime }$, and the higher one is settled as the final similarity between R1 and R2.

In actual cases, the probability that R1 is perpendicular to R2 exists when ΔRori∈[45°,135°], but in this step, only whether R1 and R2 are in the same direction or the opposite direction is it generally detected to decide whether $Mag^{2}$ or $Mag^{2}{}^{\prime }$ would be used for similarity calculation. Whether R1 and R2 are generated on a same road segment or not will be judged mainly by the magnetism similarity in the following procedures.

#### 3.3.2. Road Trace Clustering

In this step, the preprocessed road segment traces are clustered into some separate road segment clusters based on the DBSCAN algorithm. A road segment cluster is defined as a collection of users’ road segment traces whose magnetism sequences are similar to other ones in the same cluster and are dissimilar to those in other clusters. DBSCAN is a kind of density-based spatial clustering method which is commonly used in many fields [32]. In the DBSCAN algorithm, a centre-based density is defined using a distance metric, and the number of points within the settled distance metric is the density of the central point. If the density of the central point reaches a settled threshold, then it will be defined as a core point.

(1) C-neighborhood DBSCAN Clustering

For the database of user road segment traces, denoted as D; the elements in it are road segment traces to be clustered, denoted as $R_{i}$; the road segment cluster is a collection of road segment traces which are generated in a same road segment, denoted as $C_{k}$. To implement the DBSCAN algorithm to get road segment cluster $C_{k}$ from database D, the notion of *Eps-neighborhood* and values of Eps and MinPts are defined firstly.

As discussed in the above sections, we use magnetism similarity between two road segment traces to indicate the distance between two points. Since the correlation coefficient was utilized for similarity calculation between two road segments, we define a kind of correlation coefficient neighborhood (*CC-neighborhood*) for clustering.

*CC-neighborhood* of a road segment trace $R_{i}$ is defined as the road segment traces $R_{j}$ whose magnetism similarity (correlation coefficient of magnetism sequence) is within the settled range (ccRange) is termed as *CC-neighborhood* of road segment $R_{i}$ represented as $N_{CC}(R_{i})$. It is defined as following equation:

(36) $$N_{CC}(R_{i})=\{R_{j}\in D|cc(R_{i},R_{j})\in ccRange\}$$

$cc(R_{i},R_{j})$ means the magnetism sequence correlation coefficient between $R_{i}$ and $R_{j}$ using the method in Section 3.2. The ccRange is settled as [0.9,1] in this paper. Then the core road segment $R_{i}$ is defined as the one whose CC-neighborhoods are not littler than MinPts, and $R_{j}$ is directly density-reachable from $R_{i}$ respect to Eps and MinPts when $R_{j}\in N_{CC}(R_{i})$, defined as:

(37) $$\begin{array}{c} R_{j}\in N_{CC}(R_{i})\\ \left|N_{CC}(R_{i})\right|\geq MinPts \end{array}$$

In the DBSCAN algorithm, if $R_{j}$ is directly density-reachable from $R_{i}$, and $R_{k}$ is directly density-reachable from $R_{j}$, then accordingly $R_{k}$ is density-reachable from $R_{i}$, and consequently $R_{i}$, $R_{j}$ and $R_{k}$ will be collected into the same cluster. Considering the case that $R_{j}$ and $R_{k}$ are both generated in road segment RS1 and $R_{i}$ is from road segment RS2; assuming that the magnetism from $R_{j}$ are interfered with by noise which lead to a high similarity (respect to ccRange) between $R_{i}$ and $R_{j}$; then, when we carry out *CC-neighborhood* DBSCAN clustering, all the traces from road segment RS1 and RS2 would be judged to belong to the same cluster. However, if we assume that magnetism interference happens occasionally, $N_{CC}(R_{i})$ and $N_{CC}(R_{j})$ would intersect in a few of elements. On the contrary, when $N_{CC}(R_{i})$ and $N_{CC}(R_{j})$ intersect in a large amount of elements, it is probable that $R_{i}$ and $R_{j}$ are generated on the same road segment.

As discussed above, we define notion of C-*neighborhood* based on *CC-neighborhood* via adding the requirement of coincidence between $N_{CC}(R_{i})$ and $N_{CC}(R_{j})$.

*C-neighborhood* of a road segment trace $R_{i}$ is defined as: the road segment traces $R_{j}$, which is a *CC-neighborhood* of $R_{i}$ and whose *CC-neighborhood* (denoted as $N_{CC}(R_{j})$) coincide with $N_{CC}(R_{i})$ respect to a settled proportion, is termed as *C-neighborhood* of road segment $R_{i}$ represented as $N_{C}(R_{i})$. It is defined as following equation:

(38) $$\begin{array}{c} N_{C}(R_{i})=\{R_{j}\in N_{CC}(R_{i})|P(R_{i},R_{j})\geq thC\}\\ P(R_{i},R_{j})=\frac{\left|N_{CC}(R_{i})\cap N_{CC}(R_{j})\right|}{\left|N_{CC}(R_{j})\right|} \end{array}$$

$P(R_{i},R_{j})$ is coincidence proportion between $N_{CC}(R_{i})$ and $N_{CC}(R_{j})$. thC is the accepted minimum of $P(R_{i},R_{j})$. We set thC=80% in this paper.

Consequently, for the database of user road segment traces $D=\{R_{i}\}$, the road segment clusters $\left\{C_{i}\right\}$ are obtained using C-neighborhood DBSCAN Clustering by the following steps:

- All the core traces $\left\{R_{c}\right\}$ are found using C-neighborhood with respect to ccRange, thC and MinPts, represented by:
  (39) $$\begin{array}{c} R_{c}\in D\\ \left|N_{C}(R_{c})\right|\geq MinPts \end{array}$$
- Road segment traces directly density-reachable and density-reachable from one core trace $R_{c}{}^{k}$ (denoted as $\left\{R_{r}{}^{k}\right\}$) are found from database D, and a cluster $C_{k}$ is settled by:
  (40) $$\begin{array}{c} C_{k}=\{R_{c}{}^{k},R_{r}{}^{k}\}\\ C_{k}\subset D \end{array}$$
- Other clusters are found by repeating the second step, and the collection of road segment clusters are obtained finally, represented by:
  (41) $$\begin{array}{c} C=\{C_{k}\},\;k=1,2,\ldots ,K\\ C_{k}\subset D\\ \forall k_{1},k_{2}(k_{1}\neq k_{2},\;k_{1}=1,2,\ldots ,K,\;k_{2}=1,2,\ldots ,K),\;C_{k_{1}}\cap C_{k_{2}}=\emptyset \end{array}$$
  where K is the total number of clusters. Traces that couldn’t be collected to any clusters will be treated as noise and don’t participate in the following process.

Finally, the number of road segments in a floor (the number of clusters) and clusters of user traces from each road segments are obtained from the crowdsourcing database, represented by:

(42) $$\begin{array}{c} C=\{C_{1},C_{2},\ldots ,C_{K}\}\\ C_{k}=\{R_{1}^{k},R_{2}^{k},\ldots ,R_{Ck}^{k}\},k=1,2,\ldots ,K \end{array}$$

$R_{i}^{k}$ is the i-th road segment trace in the k-th cluster, and Ck is the total number of traces in the k-th cluster.

(2) Clustering Result

We collect one hundred test traces from five different corridors (road segments) using different type smartphones to test the performance of the proposed clustering algorithm. Table 2 shows the cluster result after applying *C-neighborhood DBSCAN*. The correct clustering trace is the one which is collected in the cluster in which most traces are generated in the same corridors with it. The incorrect clustering trace is the one which is collected in a cluster in which most traces are not generated in the same corridors with it.

As shown in Table 2, the correct clustering ratio is 100%, but the number of obtained clusters is more than that of corridors. Through data analysis, we found that the data in the overdetected four clusters are all acquired from one same smartphone. This result proves the good classification performance of proposed *C-neighborhood DBSCAN* algorism, and the overdetected clusters will be merged in the following procedure by connection estimation between different road segment clusters.

### 3.4. Topology Construction

In this section, traces in each cluster are merged, while connections between clusters are estimated using turns detected from the original user traces in Section 3.1, and finally, the topology map of all the road segments obtained from user traces is constructed. Moreover, topology modification is implemented to deal with errors resulting from the angle and road length estimation.

#### 3.4.1. Road Length Estimating

For road segment cluster $C_{k}=\{R_{i}^{k}\},i=1,2,\ldots ,Ck$, Ck is the total number of traces in cluster $C_{k}$. The trace with most magnetism features is chosen as the basic trace (denoted as $R_{base}^{k}$), and other traces in it are matched with $R_{base}^{k}$ using the method proposed in Section 3.2. Then we can get the zooming radio $Ratio(R_{i}^{k},R_{base}^{k})$ and distance sequence $Dis^{i}{}_{A}$ after features alignment. Finally, all the elements $R_{i}^{k}$ (except $R_{base}^{k}$) are zoomed to the same distance scale and translated to same start point (Dis=0) with $R_{base}^{k}$.

In Section 3.1, the original user trace is segmented into some straight sub-traces corresponding with different road segments, and turns connecting each sub-trace are detected and denoted as:

(43) $$I=\{R_{i},R_{j},Type,Angle\}$$

In the above procedures, $R_{i}$ and $R_{j}$ will be clustered to different road segment clusters, denoted as $R_{i}^{k1}$ and $R_{j}^{k2}$. Assuming that, for one original trace, the user’s walking state would be basically stable, then the distance scales would be same of them, represented as:

(44) $$Scale(R_{i}^{k1})=Scale(R_{j}^{k2})$$

**Figure 16:**
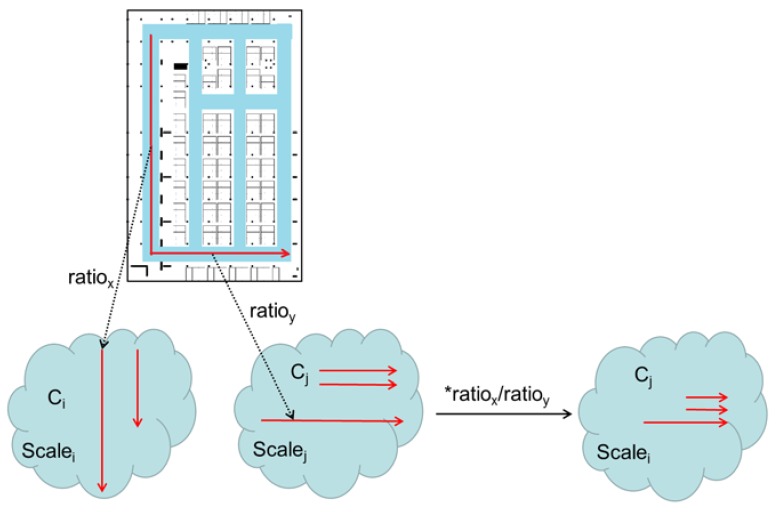
The process to make distance scale of $C_{j}$ be identical with $C_{i}$ using the connections between them.

Then it can be reckoned that:

(45) $$Scale(R_{base}^{k1})\cdot Ratio(R_{i}^{k1},R_{base}^{k1})=Scale(R_{base}^{k2})\cdot Ratio(R_{j}^{k2},R_{base}^{k2})$$

(46) $$Scale(R_{base}^{k2})=\frac{Ratio(R_{i}^{k1},R_{base}^{k1})}{Ratio(R_{j}^{k2},R_{base}^{k2})}\cdot Scale(R_{base}^{k1})$$

where Scale(*) represents the distance scale of a road segment trace. Then based on Equation (46), the distance scale of other $R_{base}^{k}$ from different road segment clusters can be set identically to $R_{base}^{k1}$ in cluster $C_{k1}$ (called the base road segment) by connected sub-traces. Furthermore, the distance sequence in each $C_{k}$ (except base road segment) would be updated to the new distance scale, denoted as $Dis^{i}{}_{B}$ for $R_{i}^{k}$ in $C_{k}$. The process is represented as Figure 16.

After that, the length of each road segment is estimated based on the same distance scale, using the following equation:

(47) $$L_{C_{k}}=\max(Dis_{B}^{i_{1}}(n))-\min(Dis_{B}^{i_{2}}(n)),\;i_{1},i_{2}=1,2,\ldots ,Ck$$

where Ck is the total number of traces in cluster $C_{k}$. $L_{C_{k}}$ is the length of the road segment cluster $C_{k}$.

#### 3.4.2. Connection Estimating Between Clusters

Utilizing turning connections I between $R_{i}$ and $R_{j}$ clustering in different clusters, the connections between different road segment clusters are found by:

(48) $$\begin{array}{l} T=\{C_{i},C_{j},P_{i},P_{j},Angle_{i,j}\}\\ Angle_{i,j}=\bar{angle(R_{x},R_{y})}\;\;,\;R_{x}\in C_{i},R_{y}\in C_{j} \end{array}$$

**Figure 17:**
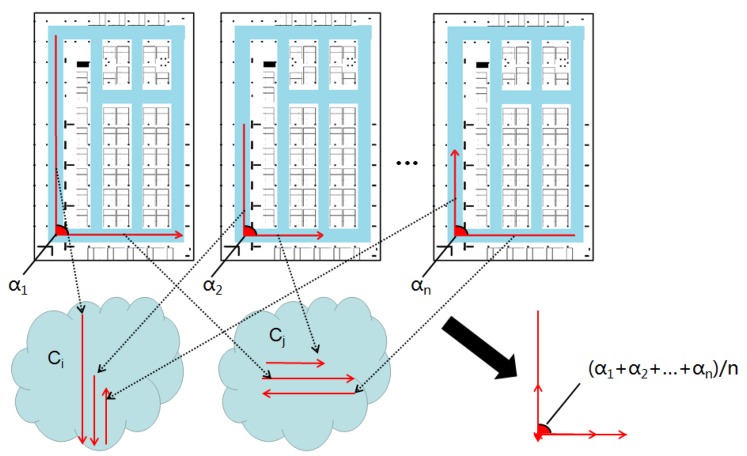
The process for estimating connection angle between clusters $C_{i}$ and $C_{j}$.

T is the connection between $C_{i}$ and $C_{j}$. $P_{i}$ and $P_{j}$ are respectively the distance identification of this connection in $C_{i}$ and $C_{j}$. $Angle_{i,j}$ is the connection angle defined as the same with connection between two different road segments trace, and valued by the mean of all the detected turning angles between $C_{i}$ and $C_{j}$. This is shown in Figure 17.

#### 3.4.3. Topology Modification and Map Construction

**Figure 18:**
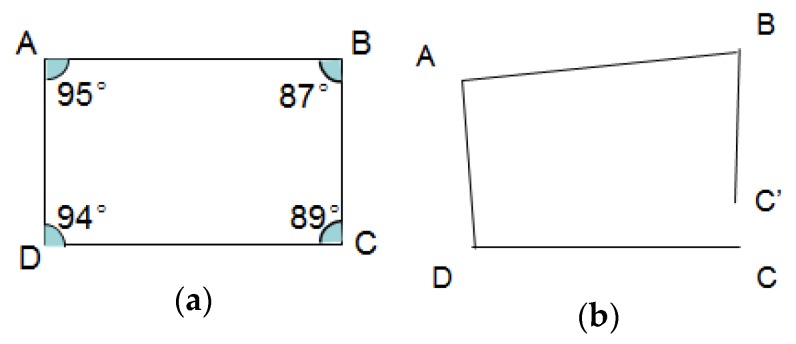
A kind of topology error caused by inaccurate angles: (**a**) a rectangle and four calculated inner angles; (**b**) the topology result of the rectangle using inaccurate inner angles. C’ and C don’t overlap.

After estimation of road segment length and connection angle of all the clusters, the topology of the road segments can be constructed. Setting the start point of base road segment (minimum of distance sequence from base road segment cluster) as (0,0) in a 2D plan, and then all the road segments can be displayed in the plan by geometry calculation. Since there should be measurement and calculation errors for lengths and angles, topology modification will be implemented to revise topology errors. The topology error is typically shown in Figure 18.

The sum of all the inner angles in a loop road is a fixed value, represented by:

(49) sum(angle)=180°·(n−2), n≥3

**Figure 19:**
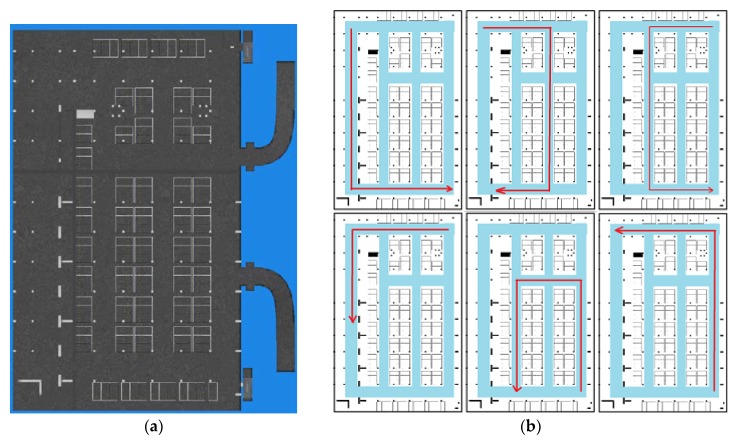
Experimental area plan and examples for crowdsourcing traces: (**a**) the floor plan of the underground parking; (**b**) parts of test traces collected by crowdsourcing users in our experiment.

Here n is the number of road segments forming the loop. In most cases, the road loop is shaped as a quadrilateral, and the sum of the inner angles is 360°. Therefore we modify the topology map using loop angle correction. As shown in Figure 19, starting with point C, the position of points D, A, B, C’ are calculated using geometry by the road segment lengths and connection angles (respectively denoted as $l_{i}$ and $\alpha_{i}$, i=1,2,…,n, n is the total number of road segments in this loop). In fact, for a loop, point C’ should be overlapped with C, so the angles $\left\{\alpha_{i}\right\}$, i=1,2,…,n will be revised to $\left\{\alpha_{i}{}^{\prime }\right\}$, i=1,2,…,n, until they can satisfy:

(50) $$\begin{array}{l} C^{\prime }=f(l_{i},{\alpha^{\prime }}_{i},C),\;i=1,2,\ldots ,n\\ \alpha_{i}^{new}={\alpha^{\prime }}_{i}|\{C^{\prime }=C,\min[\sum_{i}{\left({\alpha^{\prime }}_{i}-\alpha_{i}\right)}^{2}],\sum_{i}{\alpha^{\prime }}_{i}=180{}^{\circ}\cdot (n-2)\},\;i=1,2,\ldots ,n \end{array}$$

f(∗) indicates the geometry calculation for lengths and angles. $\left\{\alpha_{i}^{new}\right\}$, i=1,2,…,n are the modified angles that satisfied the requirement shown in Equation (50). To keep the main shape of the loop, the angle modification will be carried out in range of ±10° for each angle. Finally, the topology of the map will be modified using new angles, represented by:

(51) $$\begin{array}{l} Map=\{V,\;E\}\\ V=\left\{(x_{m},y_{m}),m=1,2,\ldots ,M\right\}\\ E=\left\{(d_{m_{1},m_{2}}),m_{1}=1,2,\ldots ,M,m_{2}=1,2,\ldots ,M\right\} \end{array}$$

V is a vector of coordinates of all the vertexes in the graph, and E is a matrix to represent the length of each edge. M is the total number of vertexes.

In addition, the map can be adjusted further, if we get the real length and orientation of one road segment in the constructed map.

What needs to be explained here is that we have adopted a simpler method for the topological modification of the map. It can reduce the complexity of the algorithm and is suitable for the layout of most indoor corridors. However, this method is only applicable to straight road segments (corridors). For curved corridors and non-channel open areas, the desired results may not be obtained. Of course, for more complex indoor scenes, we can use more indoor map information and implement detection for curved corridors to achieve better result.

### 3.5. Radio Map Construction

After final topology Map is constructed by the user traces, the 2D position coordinates of the magnetism sample points will be estimated using vertexes coordinates V and distance sequence $\left\{Dis^{i}{}_{B}\right\}$ for each road segment $R_{i}^{k}$ in cluster $C_{k}$, denoted as $Pos_{Mag}^{i}$. Because of Wi-Fi fingerprint F in the user’s road segment trace $R_{i}^{k}$ has a different sample frequency form magnetism intensity Mag, the 2D position coordinates will be interpolated linearly on each fingerprint sample time, denoted as $Pos_{F}^{i}$. So far, the Wi-Fi fingerprint collected by crowdsourcing users have been labeled by position coordinates. Since there is more than one user trace in road segment cluster $C_{k}$, in this step, we will merge RSSs collected from different crowdsourcing traces in the same road segment to generate Wi-Fi RPs which form the radio map.

#### 3.5.1. RP Generation

Considering one of the edges in the constructed map Map={V, E}, the vertexes connected by this edge are $V_{m_{1}}=(x_{m_{1}},y_{m_{1}})$ and $V_{m_{2}}=(x_{m_{2}},y_{m_{2}})$. The Wi-Fi RPs will be generated along this edge to make a grid with even distance Δd. The coordinates of RPs are calculated by:

(52) $$\begin{array}{l} \{\begin{array}{c} x_{p}=x_{m_{1}}+p\cdot \Delta d\cdot \frac{x_{m_{2}}-x_{m_{1}}}{d_{m_{1},m_{2}}}\\ y_{p}=y_{m_{1}}+p\cdot \Delta d\cdot \frac{y_{m_{2}}-y_{m_{1}}}{d_{m_{1},m_{2}}} \end{array}\\ p=1,2,\ldots ,\left\lfloor \frac{d_{m_{1},m_{2}}}{\Delta d}\right\rfloor \end{array}$$

⌊⌋ means getting a round number downward. Then all the vertexes and calculated grid points $\left(x_{p},y_{p}\right)$ for each road segment cluster constitute the RP location points in our map.

#### 3.5.2. RSS Merging on RP

For each RP generated above, we use Gaussian interpolation weights to merge Wi-Fi RSS from different user traces to the RPs locations. On RP location $\left(x_{p},y_{p}\right)$, the RSS for one of the detected Wi-Fi APs is calculated by:

(53) $$\begin{array}{l} RSS_{p}^{AP_{m}}=\sum_{n}\varpi_{p}(n)\cdot RSS^{AP_{m}}(n)\\ \varpi_{p}(n)=\frac{1}{\sqrt{2\pi }\sigma }\cdot \exp(-\frac{1}{2\sigma^{2}}\cdot [{\left(x_{n}-x_{p}\right)}^{2}+{\left(y_{n}-y_{p}\right)}^{2}]) \end{array}$$

$\left(x_{n},y_{n}\right)$ are the coordinates of the labeled fingerprint in one road segment cluster, and $RSS^{AP_{m}}(n)$ is the corresponding RSS value for the *m*-th Wi-Fi AP.

Then for the road segment cluster $C_{k}=\{R_{i}^{k}\},i=1,2,\ldots ,Ck$, the fingerprint database is acquired and represented by:

(54) $$\begin{array}{l} FD_{k}=\{(Pos^{k},F_{k})\},i=1,2,\ldots ,Ck\\ Pos^{k}=\{(x_{p},y_{p})\},F_{k}=\{fp_{p}\},p=1,2,\ldots ,P\\ fp_{p}=(RSS_{p}^{AP_{1}},RSS_{p}^{AP_{2}},\ldots ,RSS_{p}^{AP_{M}}) \end{array}$$

$FD_{k}$ is the fingerprint database for road segment cluster $C_{k}$. $\left(x_{p},y_{p}\right)$ are the RP coordinates for fingerprint $fp_{p}$. P is the number of RPs. $RSS_{p}^{AP_{m}}$ is the Wi-Fi RSS received from $AP_{m}$. M is the total number of APs which can be scanned in $C_{k}$. Finally, the fingerprint database for the whole topology Map is constructed by:

(55) $$FD=\{FD_{k}\},k=1,2,\ldots ,K$$

where FD is the whole fingerprint database for Map. K is the number of road segment clusters.

## 4. Results and Discussion

In this section we show the radio map construction result and validate its localization performance. The experiment took place in an underground parking garage of the Beijing New Technology Park of Chinese Academy of Sciences, which is covered with Wi-Fi signals (2.4 GHz). Figure 19 shows the floor plan of the underground parking and some test traces (imitating crowdsourcing user traces) used in our experiment. The crowdsourcing data are only collected in the parking area except for the entry and exit paths of the car. The size of the parking area is about 60 m × 100 m. In Figure 19b, we show the floor plan of the parking area and high light the pathway (road) in this area using blue. In order to imitate the crowdsourcing data, they are collected by four different persons, whose height and weight are shown in Table 3. Pedestrians walk along the road optionally in the experimental area and meanwhile record orientation, acceleration, angular velocity, magnetic field and Wi-Fi RSS of smartphone. The sensors data are collected using AndroSensor APP, and Wi-Fi RSSs are collected using self-developed RSSCollection APP.

Using test crowdsourcing data, the road segments are picked out and the topology map of the pathway in the experimental area is constructed through the proposed method mentioned in Section 3. Furthermore the Wi-Fi fingerprint map is constructed along each pathway. In the following content, we give the topology map construction result and Wi-Fi fingerprint localization result using the constructed Wi-Fi radio map. In addition, discussion and test result are given about the road width influence during the geomagnetism based similarity calculation of road segment.

### 4.1. Topology Map Construction Result

**Figure 20:**
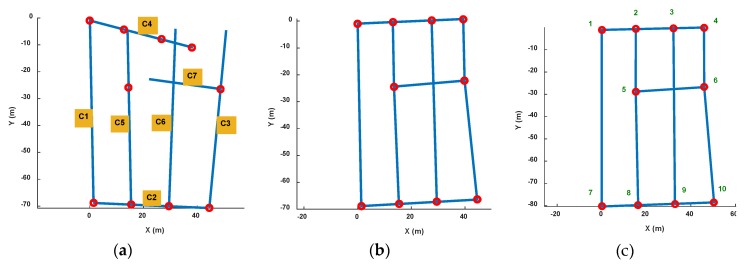
Topology map construction results: (**a**) rough result after road length and connection estimations; (**b**) pathway map after topology modification; (**c**) final pathway map after orientation correction.

Figure 20 shows the pathway map constructed by the crowdsourcing traces. Figure 20a is the rough result of the topology map after road length estimation and connection estimation between road segment clusters. The blue lines represent road segments and red points represent connections between them. Some obvious topology mistakes are shown in this result because of measurement and calculation errors for lengths and angles. Figure 20b is the pathway map after topology modification. We can get that inner angle revising makes each road segments displayed on right connection points which is alike to the real pathway in the experimental area. Figure 20c is the result after orientation and length correction of the left road segment using 0° and real road length.

In the experiment, we collected a total of 35 sets of data, and 85 sets of road segment traces are segmented from them. After calculating the magnetic field similarity and implementing the clustering algorithm, we obtained seven road clusters, which are labeled as C1~C7 in Figure 20a. It can be seen that there are topology errors in Figure 20a and the path cannot form a loop like a real road. Therefore, we then used the loop angle correction algorithm proposed in this paper to correct the topology. Because there are mainly quadrilateral roads in this testing area, we only modified the angle using quadrilateral loop in order to improve the computational efficiency. There are eight loops that used in this process, and they are [C1,C2,C3,C4], [C1,C2,C5,C4], [C1,C2,C6,C4], [C2,C3,C4,C5], [C2,C3,C4,C6], [C2,C3,C7,C5], [C2,C5,C4,C6] and [C3,C4,C5,C7].

We measure the accurate 2D coordinates of each connection points in the experiment area, and calculate the distance error of the connection points in the constructed map. Table 4 shows the distance error of each connection points (the vertex ID is shown in Figure 20c). The average distance error of the map vertex is 1.52 m. The minimum error is about 0.05 m and maximum one is 4.69 m. And the standard uncertainty is 1.4 m.

Based on the calculation result of the corner position error, we find that the position error of Vertex10 is the largest, reaching 4.68 m. In addition, the Vertex5 and Vertex9 errors also exceed 2 m. Others are below 2 m. Compared with the real indoor map, it can be seen that the error of the Vertex10 is mainly from the length estimation error of C2. Because there is no floor plan information, the road segment length is difficult to correct. Therefore, when the topology correction is performed, we only correct the connection angle so that the error of the length estimation is not eliminated. At the same time, in the loop correction, in order to obtain the final connection path, road segment length optimization is performed at the same time as the angle correction. However, in the simulation software algorithm, we extract the loop according to the list number of the detected road segment cluster. Therefore, C1 and C2 are always located at the beginning of the loop. And in loop optimization, their length is not adjusted. Then after the calibration using true length and direction of C1, the length error of C2 becomes more obvious. In addition, the test area is an underground parking, with a wide road width (more than 6 m) and a large area at each corners, which may also cause deviations in length estimations. Our algorithm does not rely on accurate indoor floor plan, but if more accurate map information can be introduced, this error can be further eliminated.

### 4.2. Radio Map Construction and Positioning Result

**Figure 21:**
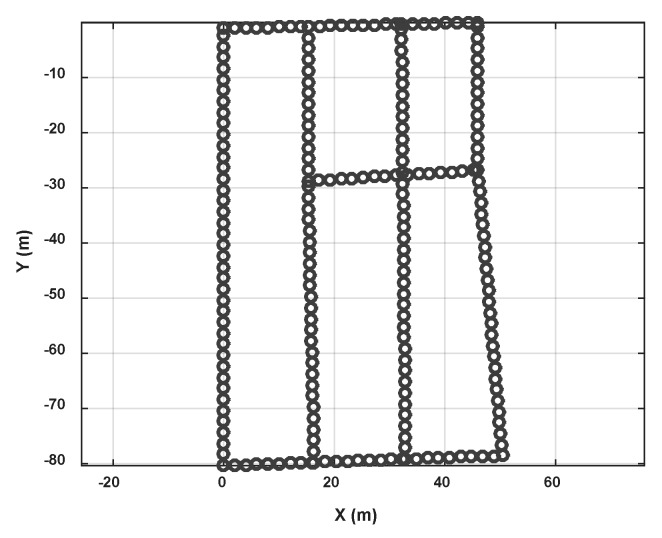
Wi-Fi RPs generated in the constructed map.

The Wi-Fi radio map are finally acquired using the constructed pathway map that is shown in Figure 21. Each point in the figure indicates RP points in the fingerprint. The distance Δd in RP grid is settled as 2 m. The parameter σ for RSS merging is 2 m.

In order to validate localization performance of the constructed Wi-Fi radio map, we pick 25 position points as test locations in the experiment area and at each test location we measure Wi-Fi RSS twice and calculate the fingerprint positioning result using the KNN algorithm (K = 3). The localization error is statistically 1.8 m (50%) and 5 m (70%), which is competitive compared with other systems based on crowdsourcing data.

Table 5 shows the comparison of our algorithm with other similar methods. These algorithms all use passive crowdsourcing user data and can provide continuous corridor localization. Zee’s reported positioning accuracy is superior to ours, but it uses a floor plan. The positioning result of RACC is similar to ours, and a floor plan is also used in this method. Our algorithm does not depend on an accurate floor plan, which makes it perform better in an unknown indoor area. PiLoc does not require a floor plan, and the authors report higher positioning accuracy than our algorithm, however, the authors’ experimental scenario is an office floor, in which the isolation of the Wi-Fi signal is better compared with the underground parking garage we used for our test. This helps the PiLoc system to form a Wi-Fi intensity distribution map with obvious features and obtain better positioning results. In addition, the PiLoc also used an optimized positioning algorithm instead of the basic KNN algorithm.

**Figure 22:**
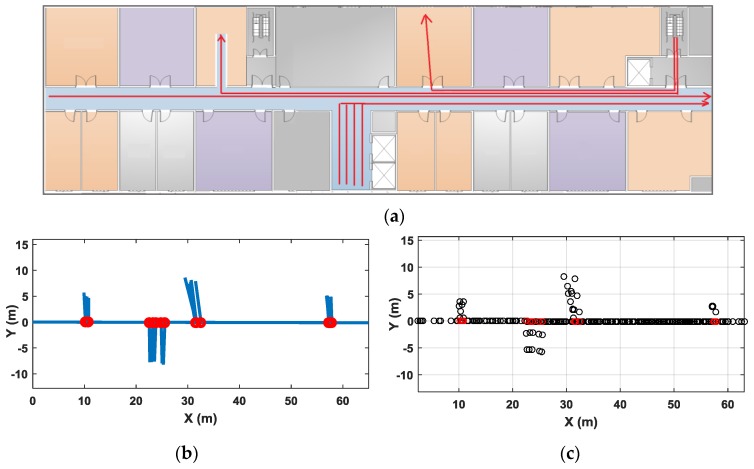
Experiment results in an office floor: (**a**) floor plan and examples for test traces; (**b**) final pathway map constructed by proposed method; (**c**) labeled Wi-Fi sample points using the constructed pathway map.

Figure 22 shows another set of test results. The test site is a floor of an office building. There is one major corridor in this area and some smaller corridors leading to the stairs, elevators and toilets. The office rooms are on two sides of the corridor. Figure 22a shows a floor plan of the experimental area, and the red lines show a part of the typical user traces in our test. Through our clustering algorithm, the major corridor in the map is identified. Because traces in small hallways or office rooms are often shorter in distance and have few magnetic features, they are not clustered into corridors in our algorithm, but they are still accurately drawn out in the constructed pathway map through their connections with the major corridor. Figure 22b shows the final pathway map after orientation correction of the major corridor. Figure 22c shows the labeled Wi-Fi sample points using coordinates of constructed map. The average positioning error in this test area is 1.7 m.

### 4.3. Road Width Influence

**Figure 23:**
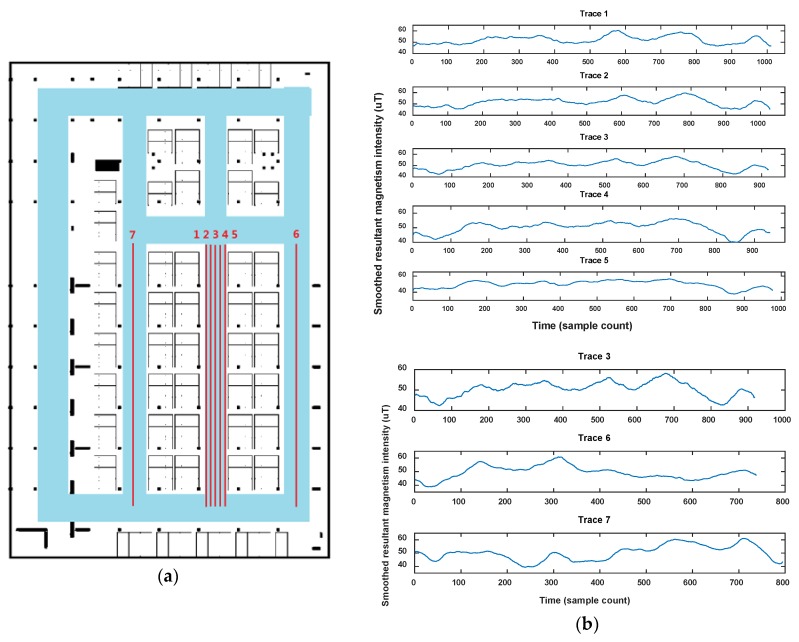
Data comparison for road width influence. (**a**) seven test traces (red lines); (**b**) the measured magnetism sequence of them.

When we use magnetism sequences to calculate road segment similarity, the road (or corridor) is abstracted as a line. However the road has a certain width in space, and when users walk along the road (corridor), these exact positions on the transverse of the road may be different from each other. To check out the influence of different transverse positions of users’ traces on the road segment similarity calculation, we collected sensor data five times on one road segment using different transverse positions and likely traces on the other road segments beside it two times, and calculated the magnetism similarity between them. Figure 23a shows the test traces (red lines, numbered from 1 to 7), and the magnetism sequence collected by the seven test traces are compared together in Figure 23b. The road width is 6 m, and the transverse interval between each trace from 1 to 5 is 1 m. It is seen in Figure 23b that the magnetism sequences have similar shape on the same road but different shapes on other roads.

Table 6 shows the similarity calculation results between each test trace. Trace 1, 2, 3, 4 and 5 show higher similarities with each other, especially with adjacent ones. On the contrary, trace 6 and 7 show lower similarity with all the other ones. We apply the C-neighborhood DBSCAN clustering proposed in this paper using the parameters as ccRange=[0.9,1], MinPts=3 and thC=50%. The algorithm obtains one cluster of {1,2,3,4,5}, and two noise traces of 6 and 7, which matches the real situation. Consequently, when we have collected abundant user data on one road segment, the road width would not impact the road segment clustering. Even if the traces on one road segment are divided into more than one cluster, these traces still have the chance to be merged together in the graph construction phase by same connections.

### 4.4. Unconstrained Smartphone Influence

In the real scenario, it would happen that a pedestrian uses his/her smartphone in different postures while walking, like messaging, calling or just holding it in the hands. The unconstrained smartphone attitude mainly affects on two factors of the proposed algorithm: one is the user’s heading and the other is the magnitude of the magnetic field, so when we using crowdsourcing user data for radio map construction in our method, these two factors would be considered:

#### 4.4.1. User heading

In order to obtain more accurate user headings, especially in the situation of unconstrained smartphone poses, it is better to use some complex algorithms to calculate the user heading, rather than use readings directly from the electronic compass. Some researchers have published relevant research results on this issue, like [30,33,34], but it is maybe still hard to estimate the exact user heading for crowdsourcing data, so during the algorithm design, we made great efforts to minimize the reliance on heading, mainly including:

(1) The proposed method uses angular velocity changes for turning detection and road segmentation, which makes it free from magnetic interference.
(2) We use the mean value of detected user headings to indicate the road segment orientation, which can partly eliminate heading errors due to local magnetic field anomalies and other short duration errors.
(3) When constructing the spatial magnetic sequence of a trace, the magnetic sampling distances are estimated only by the step count and stride length. During this period, the heading interference will not affect it, and therefore the headings will not affect the magnetic sequence similarity calculation result. In addition, the magnetic field disturbance in the indoor building would enrich the magnetic features of corridors, which is conducive to good matching and separation for indoor corridors.
(4) When constructing a pathway map, starting from the base road segment, we use the estimated lengths of the road segments and the connection angles between road segments to calculate the plane coordinates of each vertex in the map. The whole map can be further corrected if the actual orientation and length of the base road segment are known.

#### 4.4.2. Magnitude of the magnetic field

In our method, the location differentiation ability of the geomagnetic field is utilized for corridor differentiation. This means that the magnitude of the magnetic field is correspondingly different at different locations in an indoor area, but similar for adjacent locations. We use the magnitude of the magnetic field (the resultant magnetism intensity) in our method to evaluate the similarity of the user’s trajectory. The magnitude of the magnetic field is only related to the position of the smartphone rather than any rotation of the smartphone axis. When the user uses or carries the smartphone in different postures, the smartphones are in close proximity around the user body. Therefore, we speculate that under different smartphone postures, the magnitudes of magnetic field that users get are similar, and we can still use it for magnetic sequence similarity calculation.

**Figure 24:**
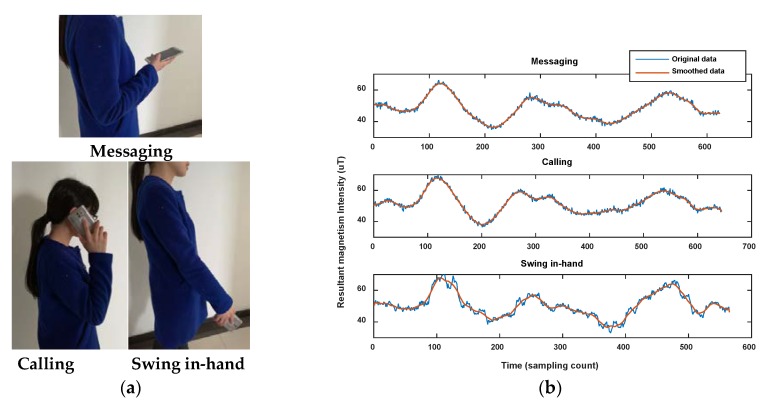
Data comparison for different smartphone attitudes. (**a**) three type of postures when a pedestrian uses smartphone; (**b**) the measured magnetism sequence of them.

Below, we collected the data of smartphone sensors in the same corridor using three typical smartphone poses, including messaging, calling and swing in-hand. We compared the magnitude of the magnetic field, and calculated the magnetic sequence similarity between each two of the three sets of data using our algorithm. Figure 24 shows magnetism sequences comparison for the three test traces. The calculated similarities between them are listed in Table 7.

Through the comparison result, we find that magnetic sequences of the three test traces show similar shape, especially when they are smoothed using a moving average filter. Most of the similarities show high values (>0.9) between them. Among them, the similarity between trace 2 (calling) and trace 3 (swing in-hand) is a bit lower (0.8632). The result proves that under different smartphone postures, we can still use the proposed algorithm for magnetic sequence similarity calculation. If a user trace can’t be clustered into any of the road segment clusters with other traces, owing to user pose complexity, it will be handled as noise and not be used for map construction.

## 5. Conclusions

In this paper, we focus on the problem of automatic Wi-Fi radio map construction using crowdsourcing data in indoor fingerprint localization systems. Based on the comparison of current systems and our analysis of the opportunities and challenges of smartphone-based indoor localization methods, we propose a geomagnetism-aided indoor radio-map construction method via passive smartphone crowdsourcing. The proposed method utilizes magnetism sequence similarity and a novel C-neighborhood DBSCAN clustering algorithm to form the pathway graph of a floor plan from crowdsourcing traces without needing an exact floor layout, and generates RPs by merging crowdsourcing Wi-Fi signal strengths to construct the radio map. The main contribution of our method include: (1) it recognizes corridors from user traces using magnetic field similarity which is relatively stable in the scenario of unconstrained smartphone use for crowdsourcing data, and also solves the problem of calculating the exact similarity between magnetism sequences when they are sampled using different walking speeds; (2) it forms the pathway graph of indoor environments using clustered road segments, and merges crowdsourcing Wi-Fi signal strengths on reference points generated along the pathway to construct the radio-map. In the designed experiments, the proposed method is proved to show good ability to construct the indoor pathway graph and Wi-Fi radio map using passive crowdsourcing data. The constructed Wi-Fi radio map can provide competitive indoor localization accuracy.

Our method is only applicable in indoor environments with obvious corridors (or roads), and a hypothesis of straight corridors (road segments) is needed in the topology modification phases. For curved corridors and non-channel open areas the desired results may not be obtained. In more complex indoor scenes, more indoor map information can be used to recognize bent corridors or open areas and they should be constructed using other suitable ways in the pathway map. That will be a focus in our future work.

## Figures and Tables

**Table 1:** Comparison of fingerprint localization methods without site surveys via passive crowdsourcing data.

Method	Floor Plan	Assistant Sensors	Reported Accuracy
WILL [22]	with	Acc.	Average 86% (room-level)
Wang [23]	with	None	95% (subarea-level)
Zee [24]	with	Acc., gyro., comp.	1.2 m (50%), 2.3 m (80%)
RACC [25]	with	Acc., gyro., comp.	1.7 m (50%), 2.2 m (80%) (fingerprint density: 1 m)
			2.9 m (50%), 4.3 m (80%) (fingerprint density: 2 m)
RCILS [26]	with	Acc., gyro., mag., bar.	Median error ~1.6 m
PiLoc [15]	without	Acc., gyro., comp.	Average 1.5 m

**Table 2:** Road segment clustering result using *C-neighborhood DBSCAN*.

Number of Test Traces	Number of Corridors	Number of Obtained Clusters	Number of Correct Clustering Traces	Number of Incorrect Clustering Traces	Correct Ratio	Incorrect Ratio
100	5	9	100	0	100%	0

**Table 3:** Different pedestrians who collected crowdsourcing data in our experiment.

	Height/cm	Weight/kg
**Pedestrian 1**	172	68
**Pedestrian 2**	168	75
**Pedestrian 3**	155	55
**Pedestrian 4**	180	75

**Table 4:** Distance errors of vertex points in the constructed pathway map.

Vertex ID	Error/m	Vertex ID	Error/m
1	1.0982	6	0.8173
2	0.7739	7	0.3158
3	1.4769	8	0.8829
4	0.0548	9	2.2865
5	2.8184	10	4.6869

**Table 5:** Results comparison with method proposed by other researchers.

Method	Floor Plan	Assistant Sensors	Reported Accuracy
Zee [24]	with	Acc., gyro., comp.	1.2 m (50%), 2.3 m (80%)
RACC [25]	with	Acc., gyro., comp.	2.9 m (50%), 4.3 m (80%)
PiLoc [15]	without	Acc., gyro., comp.	Average 1.5 m
This paper	without	Acc., gyro., comp., mag.	1.8 m (50%) and 5 m (70%)

**Table 6:** Calculated similarities between seven test traces.

	Trace 1	Trace 2	Trace 3	Trace 4	Trace 5	Trace 6	Trace 7
**Trace 1**	1	0.9577	0.8982	0.8610	0.7445	0.6791	0.6979
**Trace 2**	0.9577	1	0.9517	0.9162	0.8502	0.6595	0.7100
**Trace 3**	0.8809	0.9517	1	0.9690	0.9205	0.5916	0.7929
**Trace 4**	0.8603	0.9162	0.9690	1	0.9603	0.6744	0.6795
**Trace 5**	0.7445	0.8502	0.9205	0.9603	1	0.6261	0.6002
**Trace 6**	0.6941	0.6595	0.7118	0.6744	0.7339	1	0.6751
**Trace 7**	0.7191	0.7100	0.7829	0.7839	0.7614	0.6751	1

**Table 7:** Calculated similarities between three test traces collected using different poses.

	Trace 1	Trace 2	Trace 3
**Trace 1**	1	0.9531	0.9537
**Trace 2**	0.9531	1	0.8632
**Trace 3**	0.9537	0.8632	1
